# Repositioning Bazedoxifene as a novel IL-6/GP130 signaling antagonist for human rhabdomyosarcoma therapy

**DOI:** 10.1371/journal.pone.0180297

**Published:** 2017-07-03

**Authors:** Hui Xiao, Hemant Kumar Bid, Xiang Chen, Xiaojuan Wu, Jia Wei, Yang Bian, Chengguang Zhao, Huameng Li, Chenglong Li, Jiayuh Lin

**Affiliations:** 1 Center for Childhood Cancer and Blood Diseases, The Research Institute at Nationwide Children’s Hospital, Columbus, Ohio, United States of America; 2 Resonant Therapeutics, Inc. Life Sciences Institute, University of Michigan, Ann Arbor, Michigan, United States of America; 3 Department of Biochemistry and Molecular Biology, University of Maryland School of Medicine, Baltimore, Maryland, United States of America; 4 Department of Pediatric Surgery, Tongji Hospital, Huazhong University of Science and Technology, Wuhan, China; 5 Chemical Biology Research Center, School of Pharmaceutical Sciences, Wenzhou Medical University, University Town, Wenzhou, Zhejiang, People's Republic of China; 6 Division of Medicinal Chemistry and Pharmacognosy, College of Pharmacy, The Ohio State University, Columbus, Ohio, United States of America; 7 College of Pharmacy, the University of Florida, Gainesville, Florida, United States of America; University of South Alabama Mitchell Cancer Institute, UNITED STATES

## Abstract

Interleukins-6 (IL-6)/GP130 signaling pathway represents a promising target for cancer therapy due to its critical role in survival and progression of multiple types of cancer. We have identified Bazedoxifene, a Food and Drug Administration (FDA)-approved drug used for the prevention of postmenopausal osteoporosis, with novel function as inhibitor of IL-6/GP130 interaction. In this study, we investigate the effect of Bazedoxifene in rhabdomyosarcoma and evaluate whether inhibiting IL-6/GP130 signaling is an effective therapeutic strategy for rhabdomyosarcoma. The inhibitory effect of Bazedoxifene was assessed in rhabdomyosarcoma cell lines *in vitro* and RH30 xenograft model was used to further examine the suppressive efficacy of Bazedoxifene on tumor growth *in vivo*. Rhabdomyosarcoma cells showed their sensitivity to GP130 inhibition using gene knockdown or neutralized antibody, suggesting IL-6/GP130 as therapeutic target in rhabdomyosarcoma cells. Bazedoxifene decreased the signal transducer and activator of transcription3 (STAT3) phosphorylation, blocked STAT3 DNA binding, and down-regulated the expression of STAT3 downstream genes. Bazedoxifene also induced cell apoptosis, reduced cell viability, and inhibited colony formation in rhabdomyosarcoma cells. The inhibition of colony formation, STAT3 phosphorylation, or cell viability following Bazedoxifene treatment was partially reversed by addition of excess IL-6 or overexpression of constitutive STAT3, respectively, supporting Bazedoxifene acted through IL-6/GP130 signaling. In addition, Bazedoxifene repressed cell invasion and angiogenesis *in vitro*. Furthermore, oral administration of Bazedoxifene significantly suppressed tumor growth and expression of STAT3 phosphorylation in nude mice bearing established human rhabdomyosarcoma xenograft. Taken together, these findings validate IL-6/GP130 signaling as therapeutic target in rhabdomyosarcoma and provide first evidence that Bazedoxifene may serve as a novel promising drug targeting IL-6/GP130 for treatment of rhabdomyosarcoma.

## Introduction

Rhabdomyosarcoma is the most common pediatric soft tissue sarcoma which is aggressive and refractory [1, 2]. About 350 people are diagnosed with rhabdomyosarcoma and account for 3% of all new childhood cancers each year in the United States [3]. Significant strides in chemotherapy, surgical technique, and radiation therapy have been made in improving the outcome of patients with rhabdomyosarcoma, however, chemotherapy resistance and high rate of metastasis are still the major clinical challenges of this malignancy. Attempts to increase long time survival rate utilizing dose intensified chemotherapy regimens have not been successful, and 30–40% of pediatric cases with rhabdomyosarcoma still die of this tumor as a consequence of chemotherapy resistance [4]. In this context, identification of new therapeutic strategy is urgently needed for rhabdomyosarcoma patients.

The IL-6/GP130 signaling pathway is increasingly recognized as a pivotal player in oncogenesis, survival, and drug-resistant of various human cancers and cancer cell lines, presenting a valid drug target for cancer therapy. GP130 is the receptor subunit and receptor-associated signal transducer shared by IL-6 family of cytokines. The interaction of GP130 with IL-6–IL-6 receptor-α (IL-6Rα) binary complex to form IL-6/IL-6Rα/GP130 heterodimer, triggers the activation of several downstream signaling cascades including Janus JAK/STAT3, Ras/Raf/MEK/ERK, and PI3K/AKT pathway [5–7]. IL-6 is multifunctional cytokine with a wide range of biological activities in inflammatory, immune responses, cell apoptosis and proliferation in various cells including tumor cells [8–10]. IL-6 level is significantly elevated in cancer patients and associated with poor prognosis, survival, and metastasis [8, 11–13]. Accumulating evidence demonstrates GP130, the signal transducer of this signaling pathway, also involves in uncontrolled proliferation, resistance to apoptosis as well as metastasis in multiple cancers [14–17]. It has been reported that small molecule GP130 inhibitor SC144 shows greater potency to inhibit tumor cell growth in human ovarian cancer cell lines and xenograft model, providing the good evidence that GP130 signaling play important role in ovarian cancer progression [14]. In addition, STAT3, as the major effector of IL-6/GP130, is classified as an oncogene contributing to oncogenic transformation in cultured cells and tumor formation in nude mice [18, 19]. STAT3 is activated and translocated into nucleus to bind DNA, leading to target oncogenes transcription which participate in cancer initiating, survival, proliferation, angiogenesis, and resistance to apoptosis induced by conventional therapy [20–24]. We and others have demonstrated that persistent phosphorylated STAT3 is frequently detected in many types of human cancer including rhabdomyosarcoma and is critical for survival and tumor growth of rhabdomyosarcoma [25–27]. Another possible downstream effector of IL-6/GP130, AKT, is also activated in rhabdomyosarcoma and plays an important role in survival and oncogenesis in rhabdomyosarcoma and other types of cancer [28–32]. Therefore, inhibition of IL-6/GP130 signaling may offer new promising strategy for rhabdomyosarcoma therapy. However, to date, no small molecule inhibitor that target IL-6/GP130 signaling are available in clinical cancer therapy.

Bazedoxifene, FDA-approved drug, is used as a selective estrogen receptor α (ERα) modulator for the prevention and treatment of postmenopausal osteoporosis [33–35]. Utilizing multiple ligand simultaneous docking and drug repositioning approaches, we have identified Bazedoxifene [marketed as DUAVEE (Bazedoxifene with conjugated estrogens) by Pfizer in the prevention and treatment of postmenopausal osteoporosis] as novel inhibitor of IL-6 and GP130 protein-protein interactions [36]. In this study, we describe for the first time that Bazedoxifene with the novel function as GP130 inhibitor inhibits STAT3 phosphorylation, induces apoptosis, reduces cell viability in rhabdomyosarcoma cells, blocks angiogenesis and invasion, and suppresses the tumor growth in human rhabdomyosarcoma xenograft, suggesting that Bazedoxifene may serve as a novel therapeutic drug for rhabdomyosarcoma treatment by targeting IL-6/GP130 signaling pathway.

## Material and method

### Cell lines

Human rhabdomyosarcoma cancer cell lines (RH30, RH28, RD, and RH5) were kindly provided by Dr. Peter Houghton (Nationwide Childrens’ Hospital) in 2014, and were maintained in RPMI1640 medium supplemented with 10% FBS, 4.5 g/L L-glutamine, sodium pyruvate, and 1% penicillin/streptomycin. Human umbilical vein endothelial cells (HUVEC) were purchased from the American Type Culture Collection (ATCC) in 2014 and maintained in endothelial cell growth medium M200 (Invitrogen, Carlsbad, CA) in high glucose supplemented medium with 15% FBS, endothelial cell growth supplements (LSGS Medium, Cascade Biologics), and 2 mM glutamine. All the cell lines were resuscitated from early passage stored in liquid nitrogen and were cultured in a humidified 37°C incubator with 5% CO2.

### Reagents

IL-6, IL-4 and IFN-γ were purchased from Cell Signaling and the powder was dissolved in sterile PBS to make a 100μg/ml stock solution. Anti-GP130 neutralizing antibody was purchased from the company of R&D Systems (Gymea, NSW, Australia). Low serum growth supplement (LSGS) was obtained from Cascade Biologics Inc (Portland Oregon). Endothelial Tube formation assay kits were from Cell Biolabs, Inc. (San Diego, CA). The pre-coated Matrigel inserts for invasion assays were purchased from BD Biosciences (Palo Alto, CA). Cultrex BME Cell Invasion Assay Kit was provided from R&D Systems. The powder of Bazedoxifene was bought from company of Acesys Pharmatech and dissolved in sterile dimethyl sulfoxide (DMSO) to make a 20mM stock solution. Aliquots of the stock solution were stored at -20°C.

### Western blot analysis

Human rhabdomyosarcoma cell lines (RH30, RD, or RH28) with persistent phosphorylated STAT3 were harvested after treatment with Bazedoxifene or DMSO at 60–80% confluence overnight, then lysed in cold RIPA lysis buffer containing protease inhibitors cocktail and phosphatase inhibitor cocktail. The lysates were subjected to 10% or 12% SDS-PAGE gel and transferred to a PVDF membrane. Membranes were incubated with a 1:1000 dilution of specific primary antibody and 1:10,000 HRP conjugated secondary antibody. Primary antibodies against phosphorylated STAT3 (Tyr705, p-STAT3Y705), STAT3, cleaved caspase-3, phospho-specific ERK1/2 (Threonine 202/Tyrosine 204), P-AKT, GP130, GAPDH and secondary antibody are all from Cell Signaling Technology (Beverly, MA, USA). IL-6R antibody was purchased from Abgent (San Diego, CA, USA), and JAK1 and ER-β antibody were purchased from R&D Systems (Gymea, NSW, Australia). Membranes were analyzed using enhanced chemiluminescence plus reagents and scanned with the Storm Scanner (Amersham Pharmacia Biotech Inc, Piscataway, NJ).

### Immunoprecipitation

RD sarcoma cells were treated with Bazedoxifene 15μM for 16 hours. Cell lysates were prepared by adding lysis buffer (Cell Signaling Technology, Danvers, MA) and were pre-cleared using protein A/G agarose (Pierce Biotechnology, Rockford, IL), incubating with gentle mixing at 4°C for 2 h. GP130 was immunoprecipitated by incubating cell lysates with anti-GP130 antibody (EMD Millipore Corporation, Temecula, CA) overnight at 4°C. Protein agarose slurry was added and further incubated for 2 h at 4°C. At the end of incubation, protein A/G agarose was washed three times with IP buffer (Thermo, #28379) and proteins bound to GP130 were collected by boiling the samples in 5x loading buffer. The supernant was then separated by 10% SDS-PAGE and subjected to western blot analysis with 1:1000 dilution of primary antibodies and 1:10000 horseradish peroxidase-conjugated secondary antibodies. Antibodies against IL-6R, STAT3, GP130, and JAK1 were used for western blotting.

### Reverse transcriptase-polymerase chain reaction (RT-PCR)

Cells were treated with Bazedoxifene (10 and 20 μM) or DMSO at 60–80% confluence in the presence of 10% FBS overnight. RNA from the cells was isolated using RNeasy Kits (Qiagen) according to the manufacturer’s instruction. Reverse transcription was done using an Omniscript reverse transcription kit (Qiagen). Polymerase chain reaction (PCR) amplification was performed under the following conditions: 5 min at 94°C followed by 30 cycles of 30 seconds at 94°C, 30 sec at 53–55°C, and 60 seconds at 72°C with a final extension of 10 minutes at 72°C. Primer sequences and source information of STAT3 downstream target genes can be found in supplementary data S1 Table.

### Quantitative reverse transcriptase-PCR

Mature microRNA-21 (miR-21) and microRNA-181b (miR-181b) gene expression were measured by quantitative reverse transcriptase (qRT-PCR) [37]. Briefly, total mRNA including microRNAs was extracted from RH30 and RH28 rhabdomyosarcoma cells treated with or without Bazedoxifene using miRNeasy Mini kit (Qiagen). The cDNA was generated using the miScript II reverse transcription Kit (Qiagen). Real-Time PCR Amplification was performed using the miScript SYBR Green PCR Kit and miScript Primer Assays (Qiagen) (primer sequence of miR-21: 5' UAGCUUAUCAGACUGAUGUUGA, primer sequence of miR-181b: 5'AACAUUCAUUGCUGUCGGUGGGU) according to the manufacturer’s protocol with AppliedBiosystems 7900 HT Fast Real-Time PCR System. U6 was used as an internal control for template normalization. The threshold cycle (Ct) was set within the exponential phase of the PCR and the target PCR Ct value was normalized by subtracting the U6 Ct value, which provided the ΔCt value. The relative level of gene expression between treatments was calculated using the following equation: relative gene expression = 2^−(ΔCt sample-ΔCt control).^

### STAT3 DNA binding assay

RH30 cells were seeded in a 10-cm plate and treated with Bazedoxifene (10 and 20 μM) or DMSO overnight. The nuclear extract kit (Clontech Inc., Mountain View, CA) was used to prepare cell nuclear extracts following the manufacturer's protocol. Nuclear extracts were analyzed for STAT3 DNA binding activity using a STAT3 DNA binding ELISA kit (Active Motif, Carlsbad, CA, USA) with an ELISA-based method. Absorbance was read at 450 nm.

### Colony formation assay

The cells (RH30 and RD) were seeded and cultured for 24 hours. After pretreated with Bazedoxifene, or DMSO at 60–80% confluent for 6 hours, IL-6, IL-4, or IFN-γ (50 ng/mL) was added and incubated for 30 minutes. Cells were trypsinized and reseeded in fresh medium at 1,000 cells per 100 mm dish and allowed to grow for 2 weeks at 37°C to form colonies. The colonies were fixed with methanol and stained with crystal violet (10 g/L; Fisher Scientific, Fair Lawn, NJ, USA). Pictures of the colonies were taken using a Leica MZ 16FA inverted microscope (Leica Microsystems, Bannockburn, IL) with a 7.4 Slider Camera (Diagnostic Instruments, Inc., Sterling Heights, MI).

### MTT cell viability assay

Human rhabdomyosarcoma cells were seeded in 96-well plates at a density of 3,000 cells per well for 24 hours. The next day, cells were treated and cultured at 37°C for 48 hours. 3-(4,5-Dimethylthiazolyl)-2,5-diphenyltetrazolium bromide (MTT, Sigma) was added to each well and incubated for 4 hours. Then 150μL of N, N-dimethylformamide (Sigma) solubilization solution was added to each well to dissolve the formazan. The absorbance was read at 595 nm.

### Tansfection with constitutively active STAT3 or GP130 shRNA

RH30 cells were seeded in 6-well plate. The second day, the cells were transfected with a vector encodes the constitutive STAT3 (STAT3-C) tagged with FLAG epitope or GP130 shRNA using Lipofectamine 2000 (Invitrogen, Carlsbad, CA) according to the manufacturer’s instruction. Stable clones were obtained with G418 selection (400μg/mL) for STAT3-C transfection or with puromycin (2μg/mL) for GP130 shRNA transfection. The selected clone cells were reseeded in 96-well plates at a density of 3,000 cells per well and the next day cells were treated with or without Bazedoxifene (2.5–10 μM) for 48 hours. Cell viability was determined by MTT assay as described above.

### siRNA interfere

The human ER-β siRNA or negative control siRNA (Thermo Fisher, USA) was transfected into RD (50nM), RH28 (100nM) and RH30 (50nM) cells using Lipofectamine 2000 (Invitrogen, USA) according to the manufacturer’s instructions. After 48 hours, the cells were harvested and lysed for protein as described previously or processed to MTT cell viability assay. Western blot assay was used to detect the expression of ER-β in the transfected cells.

### Endothelial cell tube formation assay

Cell culture plates (96-well) were bottom-coated with a thin layer of ECM gel (50 μL/well), which was left to polymerize at 37°C for 60 minutes. HUVEC (2–3 × 10^4^ cells) were stimulated with VEGF in 150 μL medium and added to each well on the solidified ECM gel. Culture medium was added to each well in the presence or absence of Bazedoxifene (2.25μM). The plates were incubated at 37°C for 12–18 hours and the endothelial tubes were quantified using a fluorescent microscope after staining with Calcein AM dye. Tube forming ability was quantified by counting the total number of cell clusters and branches under a 4 × objective and four different fields per well. The results are expressed as mean fold change of branching compared with the control groups. Each experiment was performed at three times.

### Cell invasion assay

For the invasion assays, 1 × 10^5^ HUVEC cells were added into the upper chamber of the insert pre-coated with Matrigel (BD Bioscience). Cells were plated in 0% FBS medium containing 20ng/mL VEGF in the lower chamber served as chemoattractant. After several hours of incubation, the cells that did not invade through the pores were carefully wiped out with cotton wool. Then the inserts were stained with Diff Quick staining, imaged, and counted with an IX71 inverted microscope (Olympus). All the experiment was repeated 3 times independently. On the other hand, the invasion ability of RH30 and RH28 sarcoma cells treated with Bazedoxifene were evaluated using a Cultrex BME Cell Invasion Assay Kit (R&D Systems, Gymea, NSW, Australia), as described by the manufacturer’s instructions. Parental RH30 and RH28 cells were starved in serum-free medium for 24 hours prior to plating. Prior to the addition of cells, transwell chambers were coated with 1 × Basement Membrane Extract (BME) solution overnight at 37°C. Cells (5 × 10^4^ cells/chamber in serum free media) were seeded to the top chamber, and 10% FBS was added into the lower chamber. After 24 hours incubation, invasion cells on the bottom chamber were detected using fluorescence 96-well reader at 485 nm excitation and 520 nm emission. All experiments were performed in triplicate.

### Human angiogenesis array

Proteome profiler antibody based Angiogenesis array (R & D systems; Cat. No. ARY007) was used according to manufacturer’s instructions to detect the relative levels of expression of angiogenesis related proteins in control and Bazedoxifene treated cells. After blocking the membrane, 300μg of protein from untreated or Bazedoxifene (2.25μM) treated cells was added and incubated overnight at 40°C. Next day, the membrane was washed and streptavidin-HRP was added for 30 minutes. Immunoreactive signals were visualized using Super Signal Chemiluminiscence substrate (Pierce) and Biomax MR and XAR film (Eastman Kodak Co.). Array data on developed X-ray film was quantified by scanning the film using Biorad Molecular Image Gel DocTM XR+ and analyze the data using Image LabTM software.

### Mouse xenograft tumor model

All animal studies were conducted in accordance with the principles and standard procedures approved by institutional animal care and use committee of the Research Institute at Nationwide Children’s Hospital. This study was approved by the named institutional review board and ethics committee, and designed to minimize the numbers of mice used and to minimize any pain or distress. The 6-week-old female athymic nude mice were purchased from Harlan (Indianapolis, IN, USA) and maintained under barrier conditions in the animal care facility of the Research Institute at Nationwide Children’s Hospital. Human RH30 rhabdomyosarcoma cells (5 x 10^6^) in Matrigel (BD Science Franklin Lakes, NJ) were injected subcutaneously into both side flank areas of nude mice. After tumor development, mice were randomly divided into two groups consisting of 4 mice per group (8 tumors): DMSO vehicle control, gavage injection of Bazedoxifene (5mg/kg). The mice that received the same treatment were housed with four or five animals per cage. The food and water were fed with standard procedures for nude mice. The mice on study were monitored daily for signs of illness in terms of decreased activity, hunched posture, loss of appetite, loss of body weight, or lethargy. Tumor growth was determined by measuring length (L) and width (W) of the tumor every other day with a caliper. The tumor volume was calculated according to the formula: Tumor volume = 0.5236 × L × W^2^. The treatment lasted for 21 days and the mice were euthanized by CO_2_ inhalation according to the IACUC approved protocol after final Bazedoxifene treatment. The tumors were harvested from sacrificed mice, snap-frozen in liquid nitrogen and stored in -80°C. Tumors tissue homogenates were lysed and separated by SDS-PAGE to examine the expression of STAT3 phosphorylation in vehicle and Bazedoxifene treated tumors.

### Statistical analysis

Significance of correlations was done using GraphPad Prism software. Differences were analyzed with the unpaired *t* tests for comparison among two groups, or with ANOVA for multiple comparisons. Statistical analysis for mouse xenograft tumor data was performed by fitting a mixed model to conduct the repeated measures analysis. Data are presented as mean ± SD, and probability (*P*) values less than 0.05 were considered statistically significant.

## Results

### Bazedoxifene is identified as a novel GP130 inhibitor

**Fig 1:**
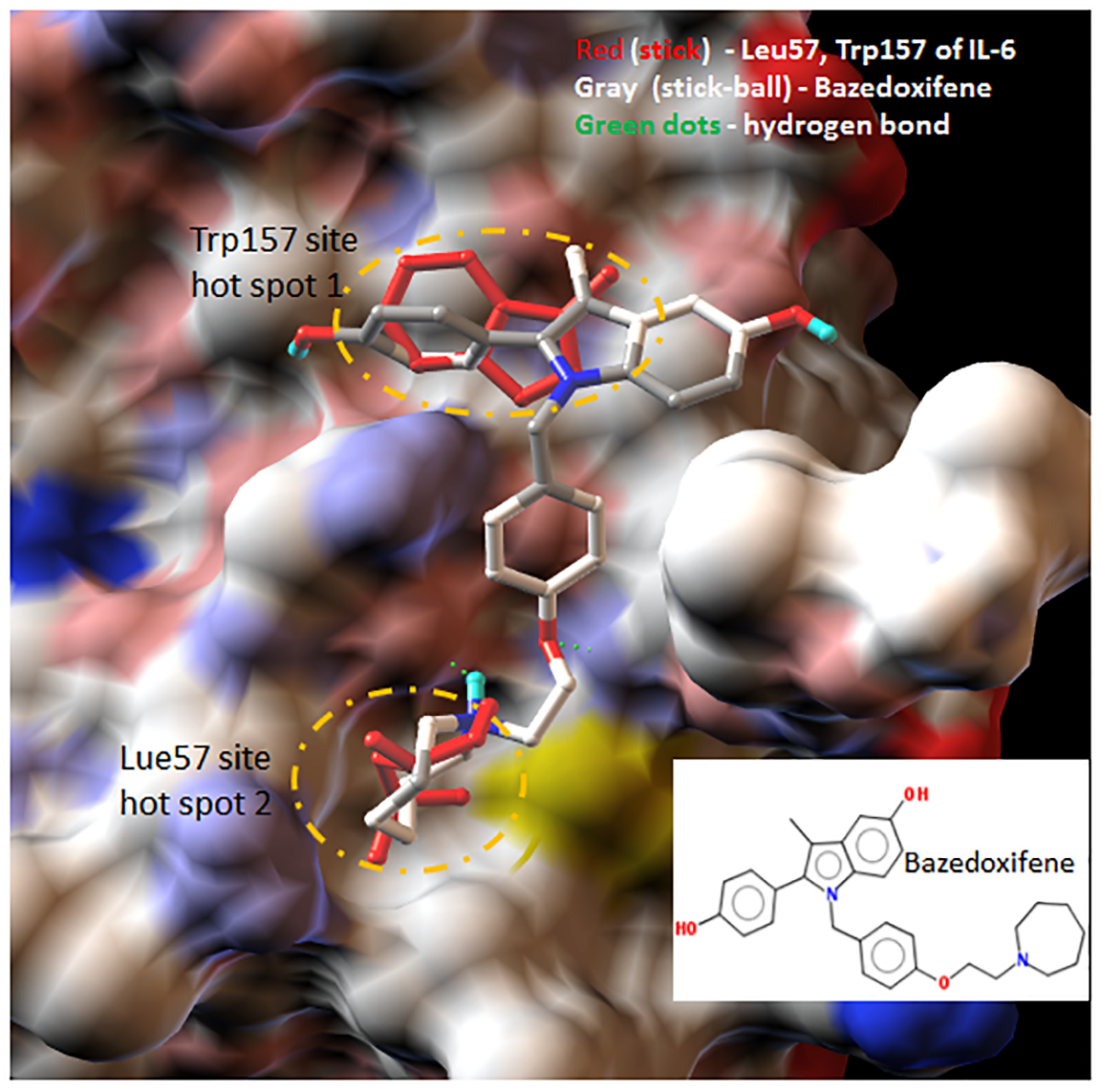
Docking modeling of drug candidate Bazedoxifene binding to GP130. Docking drug bazedoxifene (gray stick) to GP130 D1 domain shows that the indole moiety and seven-membered ring azepanyl of bazedoxifene effectively compete the native Trp157 and Leu57 binding sites (red sticks) of IL-6, respectively. Bazedoxifene also forms two hydrogen bonds with ASN92 and CYS6 of GP130 (rendered in green dot line). GP130 D1 domain (PDB code 1P9M) is represented as molecular surfaces. Picture is made using AutoDockTools (ADT).

Structure or chemical similarity search of the drug fragments mimetics of “hot spot” residues Lue57 and Trp157 of IL-6 on drug databases repositioned Bazedoxifene as a novel inhibitor of IL-6/GP130 interface. Fig 1 illustrates the docking simulation of drug candidate Bazedoxifene to GP130 D1 domain. This docking model shows that the indole moiety and seven-membered ring azepanyl of Bazedoxifene simulates the native Trp157 and Leu57 binding sites of IL-6, respectively. Bazedoxifene also forms two hydrogen bonds with ASN92 and CYS6 of GP130, both suggesting that Bazedoxifene should be able to effectively compete with IL-6 to bind to GP130. In addition, the binding of Bazedoxifene to GP130 was recently confirmed by our laboratory [36].

### Human rhabdomyosarcoma cell lines are sensitive to GP130 inhibition

**Fig 2:**
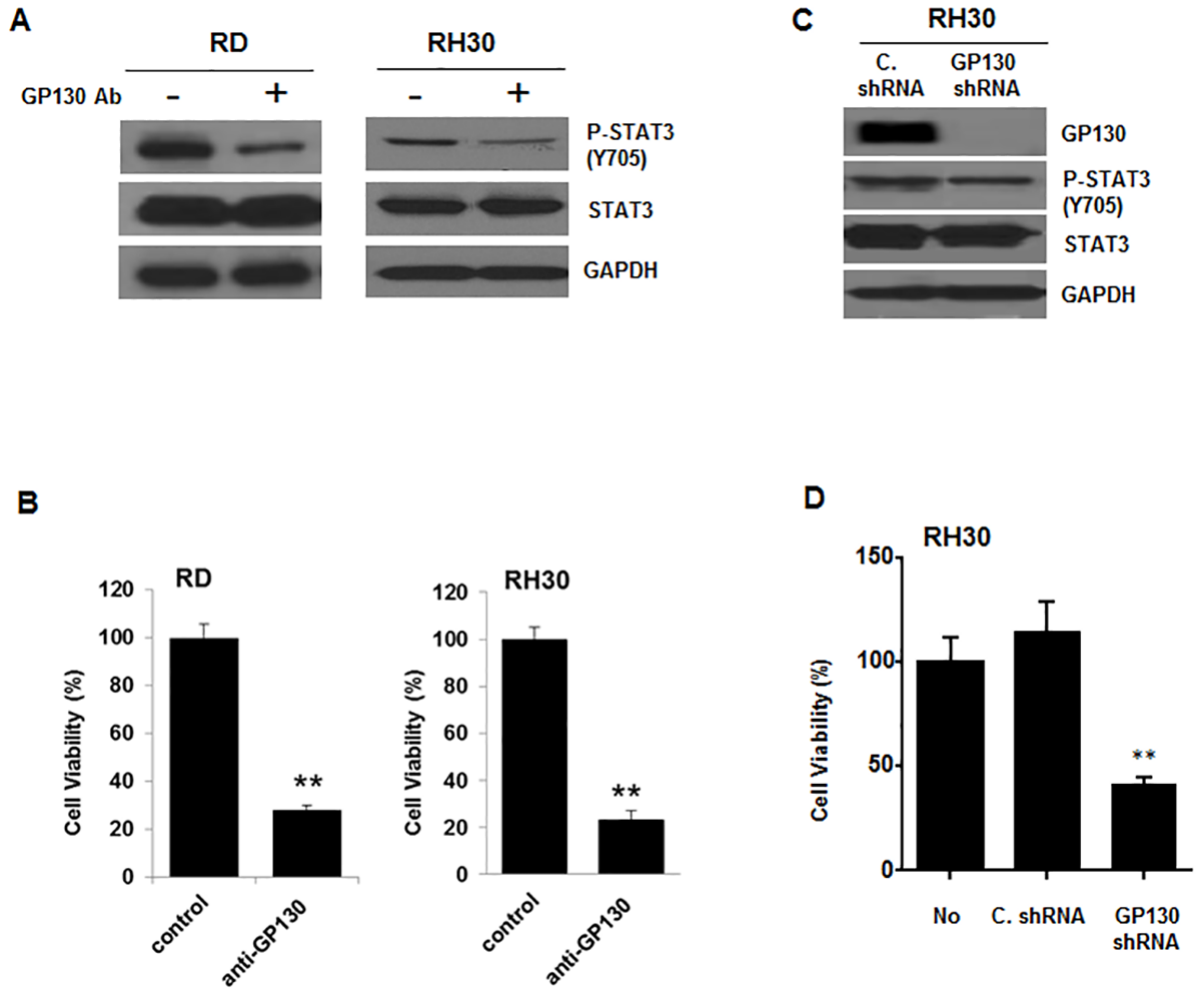
Human rhabdomyosarcoma cell lines are sensitive to GP130 inhibition. A, RH30 and RD cells were treated by neutralized anti-GP130 antibody (50 μg/ml) for 24 hours. STAT3 phosphorylation was detected by western blot. B, Cell viability was measured by MTT assay in RH30 or RD cells treated by neutralized anti-GP130 (100 μg/ml) antibody for 48 hours. The data represent mean ± SD, **, *P* < 0.01. C, GP130 and PSTAT3 (Y705) expression was evaluated by Western blot analysis in RH30 cells transfected with GP130 shRNA (C. shRNA: control shRNA). D, Cell viability was measured by MTT assay in RH30 cells transfected with GP130 shRNA (C. shRNA: control shRNA). Error bars indicate SD of mean, **, *P* < 0.01.

As the major effector of GP130, STAT3 activation has been detected in many primary rhabdomyosarcoma and rhabdomyosarcoma cell lines [26, 27, 38]. In order to study the effect of GP130 inhibition, we first examined STAT3 phosphorylation in RD and RH30 human rhabdomyosarcoma cell lines treated with an anti-GP130 antibody. As illustrated in Fig 2A, persistent phosphorylation of STAT3 (Y705) was downregulated by anti-GP130 neutralized antibody both in RD and RH30 cells, suggesting phosphorylated STAT3 is downstream of GP130 and STAT3 phosphorylation could be used as a predictive marker of GP130 inhibition. We also assessed the effect of GP130 inhibition on human rhabdomyosarcoma cell viability. The results showed that cell viability was significantly decreased in RD and RH30 cells following treatment with anti-GP130 antibody (Fig 2B). In addition, silencing of GP130 using GP130 shRNA which was confirmed by western blot analysis (Fig 2C) markedly reduced the cell viability in RH30 cells (Fig 2D). These results demonstrated that GP130 inhibition resulted in cytotoxicity in human rhabdomyosarcoma cells, which supported that IL-6/GP130 signaling plays an important role in cell survival in human rhabdomyosarcoma cells. These results also supported the rationale of inhibiting IL-6/GP130 signaling using pharmacologic drug Bazedoxifene as an effective strategy for rhabdomyosarcoma therapy.

### Bazedoxifene suppresses STAT3 phosphorylation, induces apoptosis, inhibits STAT3 DNA binding, and decreases down-stream genes expression in human rhabdomyosarcoma cells

**Fig 3:**
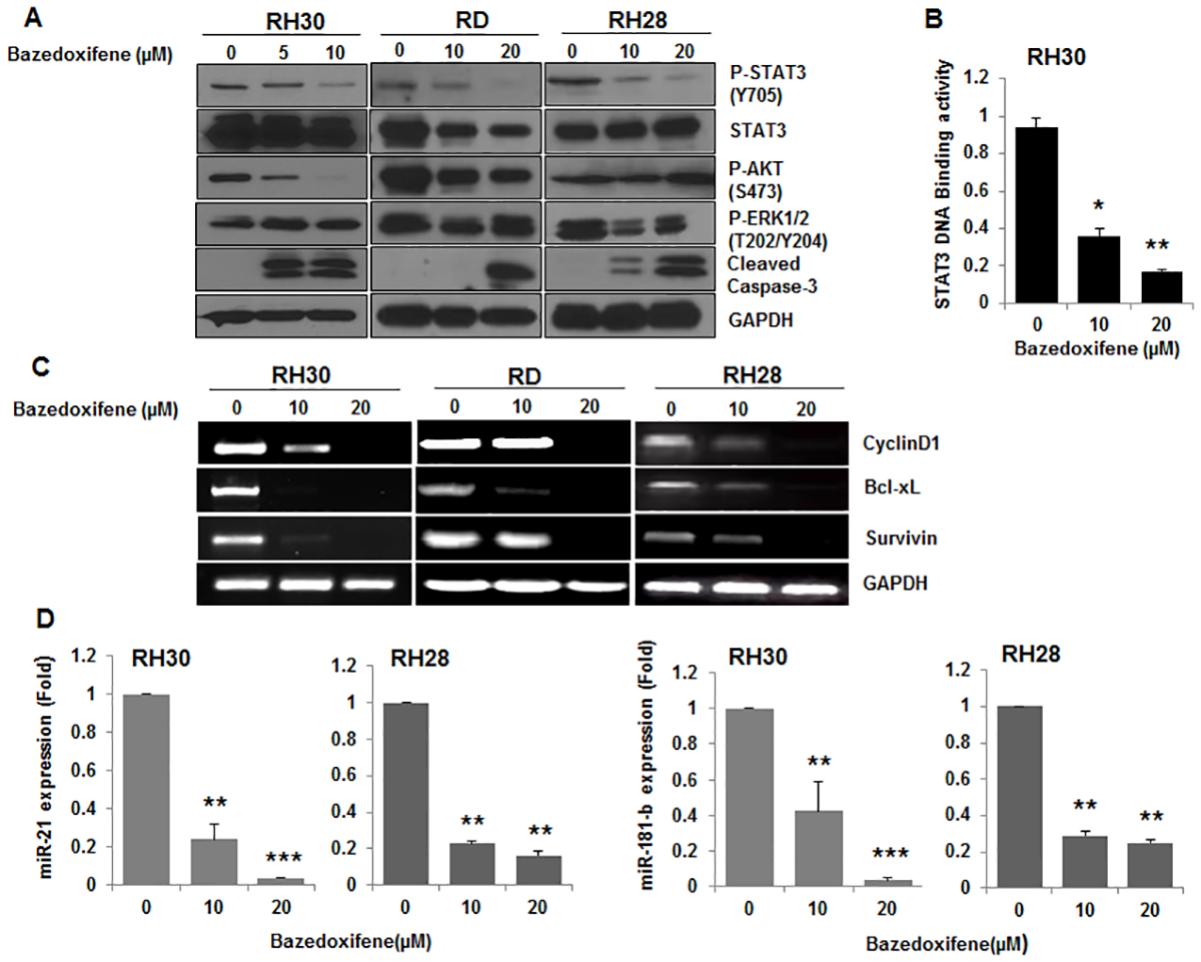
Bazedoxifene suppresses STAT3 phosphorylation, induces apoptosis, inhibits DNA binding, and decreases down-stream genes expression in human rhabdomyosarcoma cells. A, RH30, RD, and RH28 cells were treated with Bazedoxifene at the indicated concentration overnight. The protein expression of interest was determined by Western blot analysis with GAPDH as loading control. B, STAT3 DNA binding activity was measured by DNA binding assay in RH30 cells treated with Bazedoxifene (10 and 20 μM) overnight. The data represent mean ± SD, *, *P* < 0.05; **, *P* < 0.01. C, *CYCLIN D1*, *SURVIVIN*, and *BCL-XL* gene expression were detected by RT-PCR in RH30, RD, or RH28 cells treated with Bazedoxifene overnight at the indicated concentration. D, miR21 and miR-181b gene expression were analyzed by real-time quantitative RT-PCR in RH30, or RH28 cells treated with Bazedoxifene overnight at the indicated concentration, **, *P* < 0.01; ***, *P* < 0.001.

Bazedoxifene was evaluated for its inhibitory effect on IL-6/GP130/STAT3 signaling in RH30, RD, and RH28 rhabdomyosarcoma cell lines expressing elevated P-STAT3 levels. The results demonstrated that Bazedoxifene decreased the level of constitutively phosphorylated STAT3 (Y705) in all three rhabdomyosarcoma cell lines (Fig 3A). However, Bazedoxifened inhibited P-AKT in RH30 and RD cell lines, not in RH28 and only inhibited P-ERK in RH28 cells, not in RH30 and RD cell lines (Fig 3A). Bazedoxifene also exhibited inhibitory effect on STAT3 activation induced by IL-6 in RH5 rhabdomyosarcoma cells with expressing lower STAT3 phosphorylation and cultured in serum-free medium (S1 Fig). In addition, we also found in Fig 3A that Bazedoxifene treatment induced apoptosis in human rhabdomyosarcoma cells as evidenced by increasing of the cleaved caspase-3. In general, induction of apoptosis is most consistent with P-STAT3 inhibition in all three cell lines. To confirm the inhibition of STAT3 signaling by Bazedoxifene, we examined STAT3 DNA binding activity in RH30 cells treated with Bazedoxifene. As shown in Fig 3B, STAT3 DNA binding activity was significantly inhibited following Bazedoxifene treatment at the indicated concentration.

As it is known that GP130/STAT3 activation facilitated STAT3 bind to DNA to regulate the transcription of target genes including several proliferation and anti-apoptotic associated genes, so in order to further analyze the impact of Bazedoxifene on the inhibition of STAT3, we measured the expression of downstream target genes of STAT3. As shown in Fig 3C, downstream targeted genes of STAT3 such as *CYCLIN D1*, *SURVIVIN*, and *BCL-XL* in RH30, RD, and RH28 rhabdomyosarcoma cell lines were down-regulated when treated with Bazedoxifene. In addition, two STAT3 activation dependent microRNA-21(miR-21) and microRNA-181b (miR-181b), which were recently recognized oncogene implicated in multiple malignancy-related processes such as cell proliferation, anti-apoptosis, metastasis, and drug resistance [39, 40], were examined in RH30 and RH28 cells treated with Bazedoxifene using quantitative RT-PCR as described in Material and Method. We observed both miR-21 and miR-181b gene expression were dramatically reduced in RH30 and RH28 rhabdomyosarcoma cell lines by Bazedoxifene treatment (Fig 3D), which was consistent with the report that miR-21 expression was strongly suppressed by silence of STAT3 siRNA [41]. All these results further validated the inhibitory effects of Bazedoxifene on GP130 mediated STAT3 activation, suggesting the ability of Bazedoxifene to block IL-6/GP130/STAT3 signaling in rhabdomyosarcoma cells.

### Bazedoxifene-mediated inhibition is reversed by excess of IL-6 treatment or expression of constitutively active STAT3

**Fig 4:**
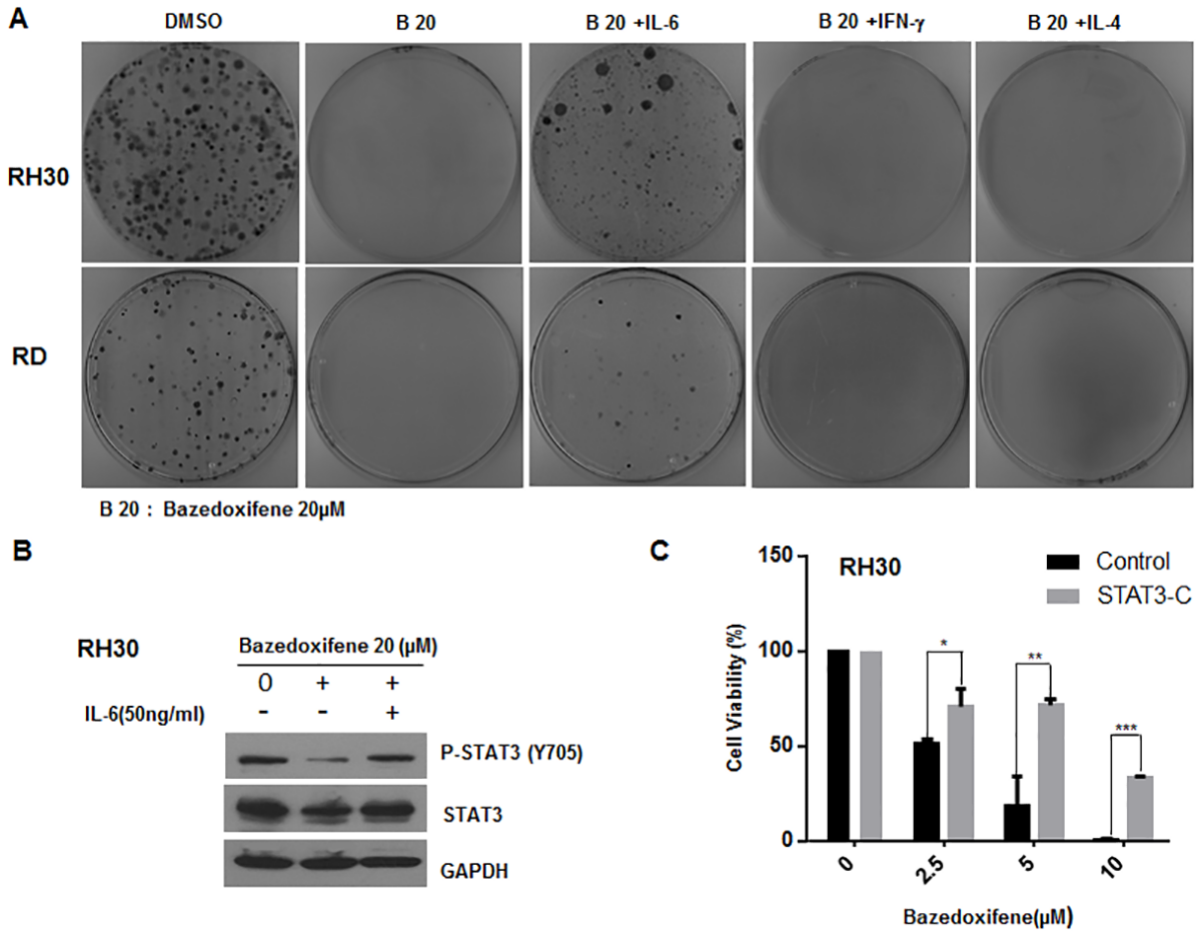
Bazedoxifene-mediated inhibition is reversed by excess of IL-6 treatment or expression of constitutively active STAT3-C. A, Colony formation assay was conducted in RH30 and RD cells as described in materials and methods. Cells were treated with Bazedoxifene (20 μM) for 6 hours, and then cultured with or without of IL-6, IFN-*γ* or IL-4 respectively for 30 minutes. Then, cells were reseeded and cultured for 2–3 weeks to grow clones. B, After treatment with Bazedoxifene (20 μM) for 6 hours, RH30 cells were stimulated with IL-6 (50 ng/ml) for 30 minutes. STAT3 phosphorylation was determined by Western blot analysis. C, RH30 cells were transfected with STAT3-C, and stable clones were selected. Selected clone cells were reseeded and treated with Bazedoxifene at indicated concentration for 48 hours. Cell viability was detected by MTT assay. Data are shown as means ±SD, *, *P* < 0.05; **, *P* < 0.01; ***, *P* < 0.001.

We next tested the ability of Bazedoxifene to inhibit colony formation and explored if excess of IL-6 treatment could reverse this inhibition using colony formation assay conducted as described in Material and Method. As observed in Fig 4A, colony formation capability of RH30 and RD cells was obviously abrogated by Bazedoxifene treatment, but adding additional IL-6 partially rescued the inhibitory effect of Bazedoxifene on colony formation. On the contrary, IFN-*γ*or IL-4 treatment was not able to affect Bazedoxifene-mediated inhibition on colony formation (Fig 4A), as IFN-*γ*and IL-4 are both non-GP130 cytokines, suggesting the inhibitory effect of Bazedoxifene is GP130 dependent. Excess of IL-6 treatment reversed the expression level of persistent STAT3 phosphorylation which is decreased by Bazedoxifene, also supporting Bazedoxifene is the inhibitor targeted IL-6/GP130 signaling (Fig 4B). In order to further examine whether Bazedoxifene-mediated inhibition was able to reversed by the expression of constitutively active STAT3 protein, RH30 cells were transfected with a constitutively active form of STAT3 (STAT3-C), and the cell viability was measured after treatment with Bazedoxifene. The result showed the cell viability in non-transfected RH30 cells was significantly reduced following Bazedoxifene treatment (at 2.5–10 μM) in a dose-dependent manner (Fig 4C). However, the cell viability decreased by Bazedoxifene treatment was partially reversed in RH30 cells which were transfected with STAT3-C expression vector (Fig 4C). In addition, to investigate if Bazedoxifene inhibited IL-6 signaling by blocking IL6/IL6R/GP130 complex information, we performed immunoprecipitation experiments and found Bazedoxifene treatment obviously blocked IL-6R binding to GP130. Furthermore, Bazedoxifene could reduce STAT3 and JAK1 binding to GP130 (S2 Fig). Taken together, these findings confirmed that Bazedoxifene acted through IL-6/GP130 signaling.

### Bazedoxifene blocks angiogenesis and invasion in *vitro*

**Fig 5:**
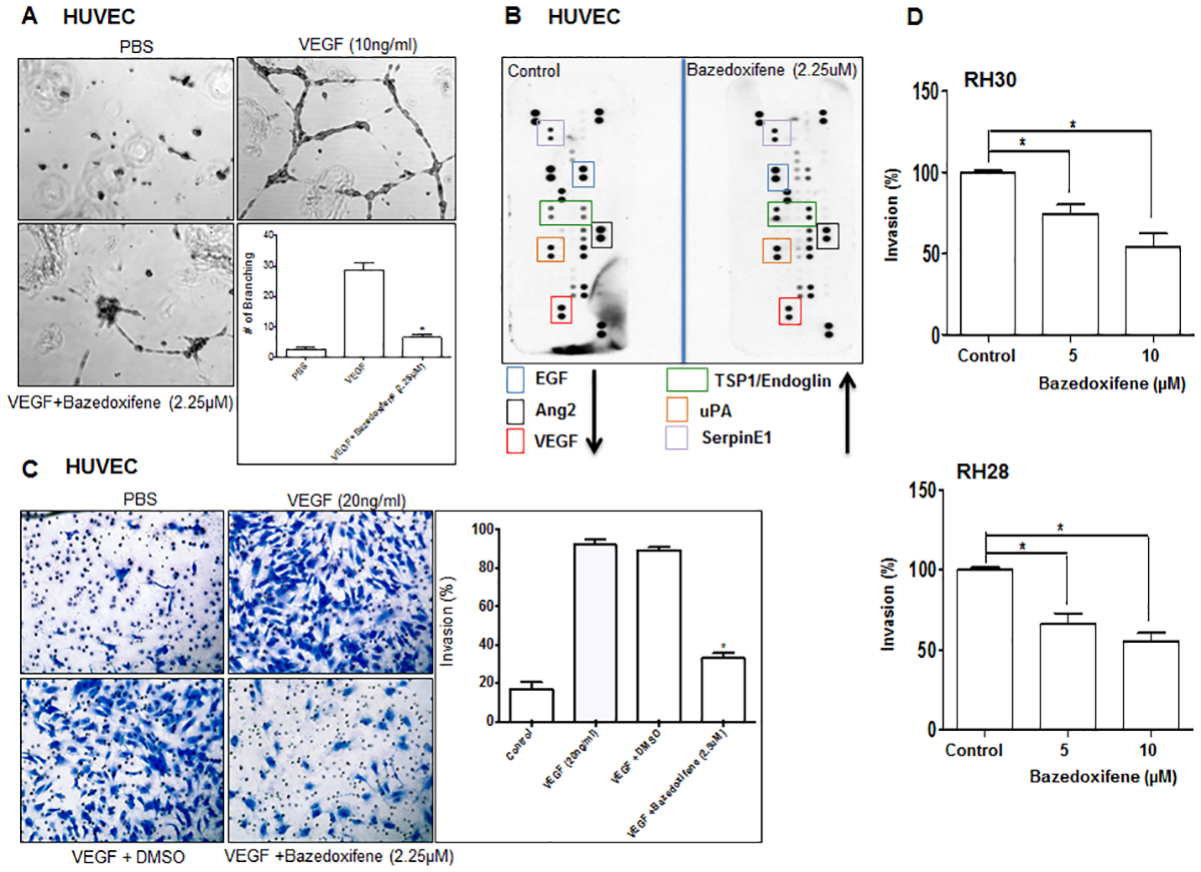
Bazedoxifene blocks angiogenesis and invasion *in vitro*. A, Bazedoxifene inhibits tube formation. HUVECs were seeded on Matrigel-coated wells with VEGF (10 ng/ml) in the absence or in the presence of Bazedoxifene and incubated for 24 hours to form a capillary network. The total number of branched tubes was then counted and representative image of capillary network formation was taken. B, Bazedoxifene downregulates several angiogenic factors *in vitro*. The proteome profiler antibody angiogenesis array was performed. The relative level of selected angiogenesis-related proteins was determined in parallel between untreated (left) and treated (right) HUVEC cells. C, Bazedoxifene inhibits endothelial cellular invasion. HUVECs (1 × 10^6^ cells/ml) were added to transwell chamber coated with Matrigel and treated with VEGF (20 ng/ml) in the absence or in presence of Bazedoxifene. After 24 hours, the number of invaded cells was counted, and results are expressed as percentage of invasion (basal invasion with no treatment). D, Bazedoxifene blocks rhabdomyosarcoma cells invasion. Parental RH30 and RH28 cells were starved in 0% FBS medium for 24 hours. After that, cells were seeded (5 × 10^4^ cells/well) to the top chamber, and 10% FBS was added into the lower chamber. Cells were treated with Bazedoxifene for 24 hours, and then invasion cells were detected in the bottom chamber using a Cultrex BME Cell Invasion Assay. Statistical analysis of three independent experiments was shown as means, *, *P* < 0.05.

Increasing evidence from functional studies indicates that GP130-mediated IL-6/STAT3 signaling activation contributes to tumor invasion and angiogenesis which are essential for tumor metastasis [42–45]. Bazedoxifene was found to disrupt IL-6 and GP130 interaction and STAT3 activation, therefore, we investigated whether Bazedoxifene blocks tumor invasion and angiogenesis. For this purpose, human HUVECs were stimulated with VEGF in the absence or presence of Bazedoxifene, and tube formation assay was first conducted as described in Material and Method section. As shown in Fig 5A, number of branches was obviously decreased by Bazedoxifene treatment, indicating Bazedoxifene inhibited formation of capillary-like structures of these endothelial cells. To further evaluate the anti-angiogenic activity of Bazedoxifene, we also examined the levels of some important angiogenic factor and angiogenesis-associate proteins using a human angiogenesis array. Compared with control group, regulators of angiogenesis including Ang2, VEGF, as well as EGF level are reduced in Bazedoxifene treatment group (Fig 5B). Furthermore, invasion assay was conducted in human HUVECs stimulated with VEGF in the absence or presence of Bazedoxifene to study the effect of Bazedoxifene on cells invasion. Fig 5C demonstrated Bazedoxifene repressed cell invasion through Matrigel coated membranes. To further examine whether Bazedoxifene could potentially decreased rhabdomyosarcoma cells invasion, we treated RH30 and RH28 cells with two different concentrations of Bazedoxifene, and found that Bazedoxifene can also significantly inhibit invasion of aggressive rhabdomyosarcoma cells (Fig 5D). All these data from the above experiments suggests that Bazedoxifene targeting IL-6/GP130/STAT3 inhibits angiogenesis and cell invasion *in vitro*.

### Bazedoxifene suppresses tumor growth of human rhabdomyosarcoma xenograft *in vivo*

**Fig 6:**
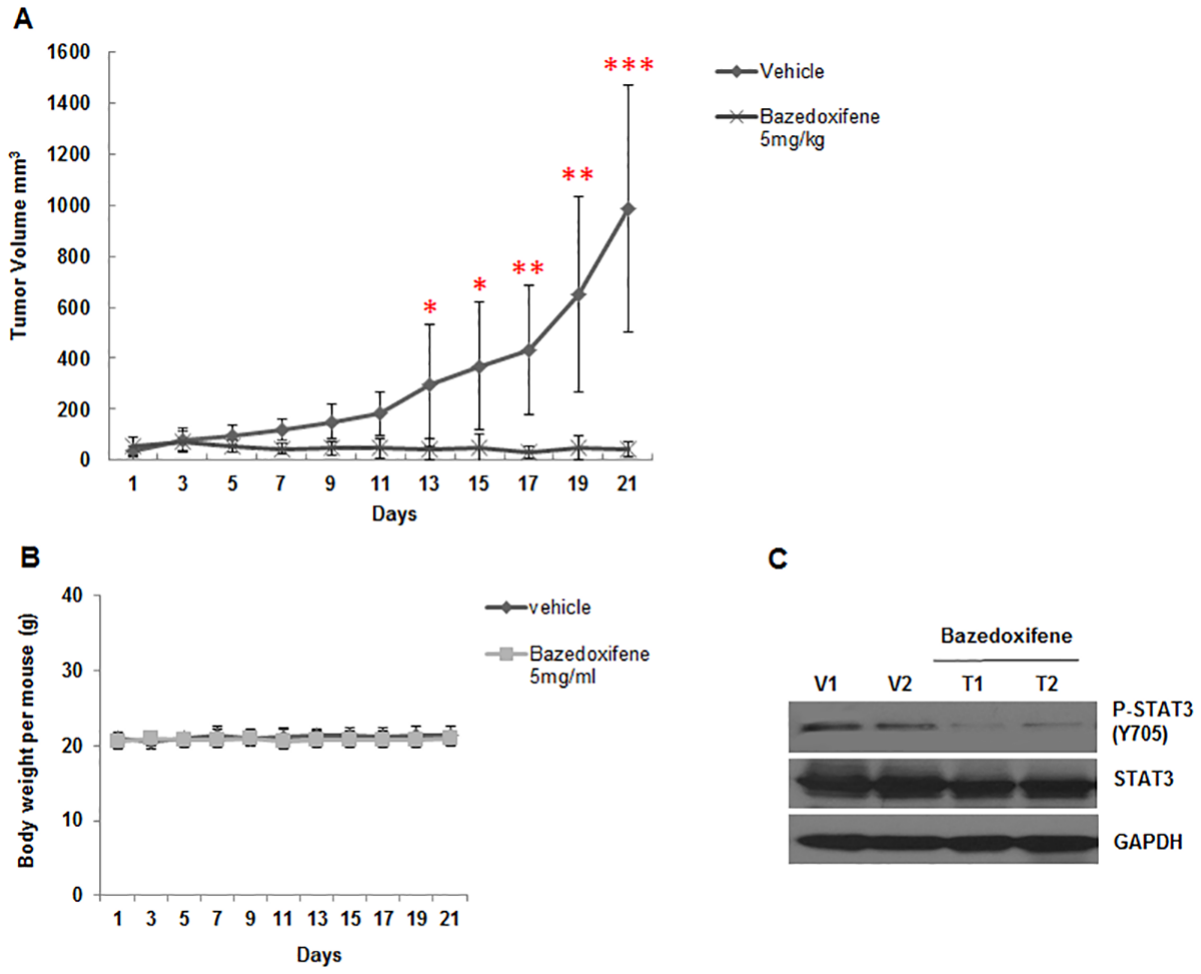
Bazedoxifene inhibits tumor growth of human rhabdomyosarcoma xenograft and STAT3 phosphorylation *in vivo*. RH30 Rhabdomyosarcoma cells (5 × 10^6^) were injected subcutaneously into nude mice. After the tumor development, vehicle or Bazedoxifene (5 mg/kg) was administered daily by oral gavage. A, Tumor size and B, Body weight were measured on the indicated days. Data represents mean ± SD, *, *P* < 0.05; **, *P* < 0.01; ***, *P* < 0.001. C, STAT3 phosphorylation from the harvested tumor tissue was determined using Western blot analysis (V: vehicle, T: tumor).

To address whether Bazedoxifene can inhibit tumor growth *in vivo*, firstly we subcutaneously implanted human RH30 rhabdomyosarcoma cells into athymic nude mice to build mouse xenograft tumor model, and then Bazedoxifene (5 mg/kg) or vehicle was given daily by oral administration after tumor development. As illustrated in Fig 6A, volume of xenograft tumor in Bazedoxifene treatment group was significantly decreased compared with the average size of tumor in vehicle control group (p < 0.001), demonstrating Bazedoxifene blocked tumor growth *in vivo*. But no obvious change in body weight (Fig 6B) or visible signs of toxicity, such as decreased activity, loss of appetite, or lethargy, was observed during the study. To detect the inhibitory effect of Bazedoxifene on activated STAT3 *in vivo*, the expression of STAT3 phosphorylation in tumor tissue was detected by Western blot. Consistent with our *in vitro* data, we found that the level of STAT3 phosphorylation in Bazedoxifene treated tumor tissue was reduced comparing to vehicle group (Fig 6C), supporting that Bazedoxifene suppressed tumor growth in nude mice through inhibition of IL-6/GP130/STAT3 signaling.

## Discussion

GP130 is emerging as attractive and promising therapeutic target for cancer therapy, because accumulating data demonstrated IL-6/GP130 signaling plays critical role in tumorigenesis, tumor proliferation, metastasis, and chemoresistance in multiple types of tumors. There are studies have documented blockade of IL-6/GP130 signaling induces apoptosis and inhibits tumor cell growth *in vitro or in vivo* [46, 47]. The neutralized antibody of IL-6 or IL-6 receptor have been tested in Phase I/II clinical trials [48–51], but it has been reported that administration of a single anti-IL-6 antibody is not efficient as expected in some clinical trials [49–51]. Madindoline A (MDL-A), a selective non-peptide antagonist to GP130 was confirmed to bind to the extracellular domain of GP130 and inhibit IL-6 dependent STAT3 phosphorylation [52]. Another GP130 inhibitor SC144 was recently reported [14]. Inhibition of GP130 by SC144 treatment decreased constitutive STAT3 phosphorylation and its downstream gene expression, induced apoptosis, as well as suppressed tumor growth in human ovarian cancer xenograft. All these studies confirmed that disrupting IL-6/GP130 signaling pathway is a potential therapeutic strategy for GP130 dependent cancer, however, until now no small molecule inhibitors that target IL-6/GP130 signaling are available for clinical cancer therapy. Therefore, it is highly desirable to identify alternative small molecule drugs to target IL-6/GP130 signaling and offer new options for anti-cancer therapy. Utilizing a novel drug discovery approach combining MLSD and drug repositioning to target GP130, we have identified the FDA-approved drug Bazedoxifene with a novel function to inhibit IL-6 and GP130 protein-protein interaction [36]. In the present study, we have for the first time demonstrated Bazedoxifene suppressed persistent STAT3 phosphorylation, decreased downstream gene expression, and induced apoptosis in human rhabdomyosarcoma cell lines expressing persistent STAT3 phosphorylation. Bazedoxifene also blocked cell invasion, repressed angiogenesis, as well as inhibited human rhabdomyosarcoma cells growth *in vitro or in vivo*.

Bazedoxifene is known as a selective estrogen receptor*α*(ER-*α* modulator which works for the prevention and treatment of osteoporosis [34, 53]. To exclude the possibility that inhibition by this drug may depend upon the inhibition of ER-*α* signaling, human rhabdomyosarcoma cell lines RH30, RD, and RH28 with constitutive STAT3 signaling that do not express ER-*α* were used in our study and most of the primary rhabdomyosarcoma tumors lack the expression of ER-*α* [54, 55]. Interestingly, knocking down ER-β with specific ER-β siRNA has no significant efficacy on the cell viability of RD, RH28, and RH30 cells, supporting that the inhibition of Bazedoxifene on rhabdomyosarcoma cells is not depend on ER-β (S3 Fig). The results that anti-GP130 antibody or GP130 shRNA treatment inhibited STAT3 phosphorylation and/or cell viability in RH30 or RD cells expressing persistent STAT3 phosphorylation were consistent with the previous discovery in ovarian cancer cell [14], indicating rhabdomyosarcoma cell lines RH30 and RD were sensitive to GP130 inhibition. As the main downstream effector of GP130 signaling pathway, STAT3 phosphorylation was abrogated by GP130 inhibition, which suggested STAT3 phosphorylation could be the predictor for the activity of GP130 inhibitor. The fact that Bazedoxifene significantly decreased STAT3 phosphorylation *in vitro or in vivo* and repressed STAT3 DNA binding activity in rhabdomyosarcoma cells suggested its potency. The reason that Bazedoxifene didn’t inhibited P-AKT or P-ERK in all three rhabdomyosarcoma cell lines could be the activation of P-AKT or P-ERK not only depend on IL-6/GP130 signaling, but also depend on the stimulation of others cytokines or growth factor. The expression of several known GP130/STAT3 downstream target genes and microRNA such as *CYCLIN D1*, *SURVIVIN*, and *BCL-XL*, miR-21, and miR-181b was reduced following Bazedoxifene treatment shown by RT-PCR or quantitative RT-PCR analysis, which also support the idea that Bazedoxifene is a potent inhibitor of GP130. Bazedoxifene showed its anti-tumor activity by inducing apoptosis in rhabdomyosarcoma cells due to IL-6/GP130 signaling inhibition. The fact that Bazedoxifene inhibited cancer cell proliferation and blocked cancer cell colony formation gave more supports for its activity. Furthermore, the result that oral administration of Bazedoxifene suppressed human rhabdomyosarcoma growth in a mouse xenograft model provided further confirmation for the anti-tumor ability of Bazedoxifene.

The computational simulation analysis of molecular structure of Bazedoxifene has found that Bazedixifene could bind to GP130 to block IL-6/GP130 signaling. Bazedoxifene fragment perfectly overlapped with the key interacting residues of IL-6 in the binding interface of IL-6/GP130 shown by docking modeling of Bazedoxifene to GP130 D1 domain. This suggested Bazedoxifene could compete with IL-6 binding to GP130 and therefore disrupt IL-6/GP130 signaling. The direct binding of Bazedoxifene to GP130 indicated by drug affinity responsive target stability (DARTS) assay in our previous report has confirmed this suggestion [36]. The further confirmation of Bazedoxifene targeting IL-6/GP130 comes from inhibitory effect of Bazedoxifene on cell assays. The fact that excess of IL-6 treatment partially reversed the persistent STAT3 phosphorylation and colony formation decreased by Bazedoxifene, which suggested Bazedoxifene targets GP130, consistent with the prediction derived from computational simulation analysis of Bazedoxifene molecular structure. We also observed overexpression of constitutively active STAT3 protein rescued the cell viability of human rhabdomyosarcoma cells reduced by Bazedoxifene, providing more supportive evidence for the suggestion that Bazedoxifene acts through IL-6/GP130 signaling pathway. Furthermore, we verified that Bazedoxifene could block GP130 to interact with IL-6R, JAK1, and STAT3, and thus inhibit IL6/IL6R/GP130 signaling to JAK1/STAT3.

In clinical trials, FDA approved drug Bazedoxifene is commonly used as a selective estrogen receptor modulator for osteoporosis prevention and treatment in postmenopausal women. As third generation estrogen receptor modulator, the selectivity and safety of Bazedoxifene is improved over Tamoxifen whose major adverse effect is increasing the incidence of uterine cancer [33, 56, 57]. In phase III clinical studies, Bazedoxifene showed a favorable reproductive (endometrial, ovarian, and breast) safety profile in postmenopausal women over three [58] and seven [59] years respectively. In present study, while Bazedoxifene showed its suppressive efficacy on tumor growth in mouse xenograft model *in vivo*, we did not see any obvious side effect in terms of decreased activity, loss of appetite, loss of body weight, or lethargy, indicating a safety profile of Bazedoxifene treatment. In general, basing on our or others experiments, Bazedoxifene as a monotherapy or combinational treatment seems to be all well tolerated [53, 59]. In addition, as mentioned above, Bazedoxifene did not increase the incidence of cancer, on the contrary, it has been reported Bazedoxifene exhibits anti-proliferation activity against human breast cancer cells [60, 61], but the molecular mechanism is not clear. The fact of blocking IL-6/GP130 signaling by Bazedoxifene could give explanation for this. On the basis of definite anti-tumor efficacy of Bazedoxifene and its safety, Bazedoxifene may be extended application to investigate its inhibitory effect on other cancer types that persistent express IL-6/GP130 signaling.

In conclusion, we provide evidence that Bazedoxifene is a novel inhibitor of IL-6/GP130 signaling. Bazedoxifene effectively blocks constitutive STAT3 activation, induces apoptosis, and inhibits human rhabdomyosarcoma cells growth *in vitro* or *in vivo*, offering an attractive therapeutic approach to rhabdomyosarcoma. As FDA-approved drug with known pharmacokinetics and safety, Bazedoxifene has great potential to be used in clinic to benefit patients with IL-6/GP130 dependent cancers.

## Supporting information

S1 Fig Bazedoxifene decreased STAT3 phosphorylation induced by IL-6 in rhabdomyosarcoma cells. RH5 rhabdomyosarcoma cells with lower P-STAT3 expression was cultured in FBS free media for 24 hours. Cells were pretreated with Bazedoxifene at the indicated concentration for 8 hours, and then IL-6 (50ng/ml) was added for 30 minutes. The expression level of P-STAT3 (Y705) was assessed by Western blot analysis with GAPDH as loading control.
(PPTX)

S2 Fig Bazedoxifene blocks GP130 to interact with IL-6R and JAK1/STAT3. RD rhabdomyosarcoma cells were treated with Bazedoxifene 15μM for 16 hours. Then cell lysates were subjected to immunoprecipitation and the expression level of GP130, IL-6R, JAK1, and STAT3 was evaluated using Western blot analysis with GAPDH as loading control.
(PPTX)

S3 Fig Knocking down ER-β with specific ER-β siRNA has no significant efficacy on the cell viability of RD, RH28, and RH30. The human ER-β siRNA or negative control siRNA was transfected into RD (50nM), RH28 (100nM) and RH30 (50nM) cells using Lipofectamine2000. A, Western blot assay was used to detect the expression of ER-β in the transfected cells to confirm the transfection efficacy. B, MTT assay was conducted to detect cell viability of the transfected rhabdomyosarcoma cells.
(PPTX)

S1 Table The DNA sequences of primers of STAT3 downstream target genes (Cyclin D1, Survivin, Bcl-xl and GAPDH) used for RT-PCR analysis. (PPTX)

S1 File The animal experiment data. (XLSX)

## References

[1] Pappo A. Rhabdomyosarcoma and other soft tissue sarcomas in children. Curr Opin Oncol 1996;8:311–6. 8869806 10.1097/00001622-199607000-00008

[2] Siegel DA, King J, Tai E, Buchanan N, Ajani UA, Li J. Cancer incidence rates and trends among children and adolescents in the United States, 2001–2009. Pediatrics. 2014;134(4):e945–55. Epub 2014/09/10. doi: 10.1542/peds.2013-3926 ; 25201796 10.1542/peds.2013-3926 PMC4536809

[3] Egas-Bejar D, Huh WW. Rhabdomyosarcoma in adolescent and young adult patients: current perspectives. Adolescent health, medicine and therapeutics. 2014;5:115–25. Epub 2014/06/27. doi: 10.2147/AHMT.S44582 ; 24966711 10.2147/AHMT.S44582 PMC4069040

[4] Arndt CA, Rose PS, Folpe AL, Laack NN. Common musculoskeletal tumors of childhood and adolescence. Mayo Clinic proceedings. 2012;87(5):475–87. Epub 2012/05/09. doi: 10.1016/j.mayocp.2012.01.015 ; 22560526 10.1016/j.mayocp.2012.01.015 PMC3538469

[5] Kishimoto T. Signal transduction through homo- or heterodimers of gp130. Stem Cells. 1994;1:37–44. 7696968

[6] Murakami M, Hibi M, Nakagawa N, Nakagawa T, Yasukawa K, Yamanishi K, et al IL-6-induced homodimerization of gp130 and associated activation of a tyrosine kinase. Science. 1993;260(5115):1808–10. Epub 1993/06/18. . 8511589 10.1126/science.8511589

[7] Narazaki M, Witthuhn B, Yoshida K, Silvennoinen O, Yasukawa K, Ihle J, et al Activation of JAK2 kinase mediated by the interleukin 6 signal transducer gp130. Proc Natl Acad Sci U S A. 1994;91:2285–9. 8134389 10.1073/pnas.91.6.2285 PMC43355

[8] Hodge DR, Hurt EM, Farrar WL. The role of IL-6 and STAT3 in inflammation and cancer. Eur J Cancer. 2005;41(16):2502–12. doi: 10.1016/j.ejca.2005.08.016 . 16199153 10.1016/j.ejca.2005.08.016

[9] Neurath MF, Finotto S. IL-6 signaling in autoimmunity, chronic inflammation and inflammation-associated cancer. Cytokine & growth factor reviews. 2011;22(2):83–9. Epub 2011/03/08. doi: 10.1016/j.cytogfr.2011.02.003 . 21377916 10.1016/j.cytogfr.2011.02.003

[10] Naugler WE, Karin M. The wolf in sheep's clothing: the role of interleukin-6 in immunity, inflammation and cancer. Trends in molecular medicine. 2008;14(3):109–19. Epub 2008/02/12. doi: 10.1016/j.molmed.2007.12.007 . 18261959 10.1016/j.molmed.2007.12.007

[11] Bachelot T, Ray-Coquard I, Menetrier-Caux C, Rastkha M, Duc A, Blay JY. Prognostic value of serum levels of interleukin 6 and of serum and plasma levels of vascular endothelial growth factor in hormone-refractory metastatic breast cancer patients. British journal of cancer. 2003;88(11):1721–6. doi: 10.1038/sj.bjc.6600956 ; 12771987 10.1038/sj.bjc.6600956 PMC2377148

[12] Andrews B, Shariat SF, Kim JH, Wheeler TM, Slawin KM, Lerner SP. Preoperative plasma levels of interleukin-6 and its soluble receptor predict disease recurrence and survival of patients with bladder cancer. The Journal of urology. 2002;167(3):1475–81. Epub 2002/02/08. . 11832773

[13] Chen MF, Chen PT, Lu MS, Lin PY, Chen WC, Lee KD. IL-6 expression predicts treatment response and outcome in squamous cell carcinoma of the esophagus. Molecular cancer. 2013;12:26 Epub 2013/04/09. doi: 10.1186/1476-4598-12-26 ; 23561329 10.1186/1476-4598-12-26 PMC3667147

[14] Xu S, Grande F, Garofalo A, Neamati N. Discovery of a novel orally active small-molecule gp130 inhibitor for the treatment of ovarian cancer. Molecular cancer therapeutics. 2013;12(6):937–49. Epub 2013/03/29. doi: 10.1158/1535-7163.MCT-12-1082 . 23536726 10.1158/1535-7163.MCT-12-1082

[15] Rebouissou S, Amessou M, Couchy G, Poussin K, Imbeaud S, Pilati C, et al Frequent in-frame somatic deletions activate gp130 in inflammatory hepatocellular tumours. Nature. 2009;457(7226):200–4. Epub 2008/11/21. doi: 10.1038/nature07475 ; 19020503 10.1038/nature07475 PMC2695248

[16] Jones SA, Scheller J, Rose-John S. Therapeutic strategies for the clinical blockade of IL-6/gp130 signaling. The Journal of clinical investigation. 2011;121(9):3375–83. Epub 2011/09/02. doi: 10.1172/JCI57158 ; 21881215 10.1172/JCI57158 PMC3163962

[17] Xu S, Neamati N. gp130: a promising drug target for cancer therapy. Expert opinion on therapeutic targets. 2013;17(11):1303–28. Epub 2013/10/09. doi: 10.1517/14728222.2013.830105 . 24099136 10.1517/14728222.2013.830105

[18] Bromberg JF, Wrzeszczynska MH, Devgan G, Zhao Y, Pestell RG, Albanese C, et al Stat3 as an oncogene. Cell. 1999;98(3):295–303. . 10458605 10.1016/s0092-8674(00)81959-5

[19] Schlessinger K, Levy DE. Malignant transformation but not normal cell growth depends on signal transducer and activator of transcription 3. Cancer research. 2005;65(13):5828–34. Epub 2005/07/05. doi: 10.1158/0008-5472.CAN-05-0317 ; 15994959 10.1158/0008-5472.CAN-05-0317 PMC2100417

[20] Bromberg J, Darnell JE Jr. The role of STATs in transcriptional control and their impact on cellular function. Oncogene. 2000;19(21):2468–73. Epub 2000/06/13. doi: 10.1038/sj.onc.1203476 . 10851045 10.1038/sj.onc.1203476

[21] Turkson J, Jove R. STAT proteins: novel molecular targets for cancer drug discovery. Oncogene. 2000;19(56):6613–26. doi: 10.1038/sj.onc.1204086 . 11426647 10.1038/sj.onc.1204086

[22] Wei LH, Kuo ML, Chen CA, Chou CH, Lai KB, Lee CN, et al Interleukin-6 promotes cervical tumor growth by VEGF-dependent angiogenesis via a STAT3 pathway. Oncogene. 2003;22(10):1517–27. Epub 2003/03/12. doi: 10.1038/sj.onc.1206226 . 12629515 10.1038/sj.onc.1206226

[23] Gritsko T, Williams A, Turkson J, Kaneko S, Bowman T, Huang M, et al Persistent activation of stat3 signaling induces survivin gene expression and confers resistance to apoptosis in human breast cancer cells. Clinical cancer research: an official journal of the American Association for Cancer Research. 2006;12(1):11–9. Epub 2006/01/07. doi: 10.1158/1078-0432.CCR-04-1752 . 16397018 10.1158/1078-0432.CCR-04-1752

[24] Real PJ, Sierra A, De Juan A, Segovia JC, Lopez-Vega JM, Fernandez-Luna JL. Resistance to chemotherapy via Stat3-dependent overexpression of Bcl-2 in metastatic breast cancer cells. Oncogene. 2002;21(50):7611–8. Epub 2002/10/26. doi: 10.1038/sj.onc.1206004 . 12400004 10.1038/sj.onc.1206004

[25] Chen C, Loy A, Cen L, Chan C, Hsieh F, Cheng G, et al Signal transducer and activator of transcription 3 is involved in cell growth and survival of human rhabdomyosarcoma and osteosarcoma cells. BMC cancer. 2007;7:111 doi: 10.1186/1471-2407-7-111 17598902 10.1186/1471-2407-7-111 PMC1964761

[26] Liu A, Liu Y, Xu Z, Yu W, Wang H, Li C, et al Novel small molecule, XZH-5, inhibits constitutive and interleukin-6-induced STAT3 phosphorylation in human rhabdomyosarcoma cells. Cancer science. 2011;102(7):1381–7. Epub 2011/03/26. doi: 10.1111/j.1349-7006.2011.01932.x . 21435102 10.1111/j.1349-7006.2011.01932.x PMC11158026

[27] Yan S, Li Z, Thiele CJ. Inhibition of STAT3 with orally active JAK inhibitor, AZD1480, decreases tumor growth in Neuroblastoma and Pediatric Sarcomas In vitro and In vivo. Oncotarget. 2013;4(3):433–45. Epub 2013/03/28. doi: 10.18632/oncotarget.930 ; 23531921 10.18632/oncotarget.930 PMC3717306

[28] Tomita Y, Morooka T, Hoshida Y, Zhang B, Qiu Y, Nakamichi I, et al Prognostic significance of activated AKT expression in soft-tissue sarcoma. Clinical cancer research: an official journal of the American Association for Cancer Research. 2006;12(10):3070–7. Epub 2006/05/19. doi: 10.1158/1078-0432.CCR-05-1732 . 16707604 10.1158/1078-0432.CCR-05-1732

[29] Renshaw J, Taylor KR, Bishop R, Valenti M, De Haven Brandon A, Gowan S, et al Dual blockade of the PI3K/AKT/mTOR (AZD8055) and RAS/MEK/ERK (AZD6244) pathways synergistically inhibits rhabdomyosarcoma cell growth in vitro and in vivo. Clinical cancer research: an official journal of the American Association for Cancer Research. 2013;19(21):5940–51. Epub 2013/08/07. doi: 10.1158/1078-0432.CCR-13-0850 ; 23918606 10.1158/1078-0432.CCR-13-0850 PMC3818134

[30] Petricoin EF 3rd, Espina V, Araujo RP, Midura B, Yeung C, Wan X, et al Phosphoprotein pathway mapping: Akt/mammalian target of rapamycin activation is negatively associated with childhood rhabdomyosarcoma survival. Cancer research. 2007;67(7):3431–40. Epub 2007/04/06. doi: 10.1158/0008-5472.CAN-06-1344 . 17409454 10.1158/0008-5472.CAN-06-1344

[31] Cen L, Hsieh FC, Lin HJ, Chen CS, Qualman SJ, Lin J. PDK-1/AKT pathway as a novel therapeutic target in rhabdomyosarcoma cells using OSU-03012 compound. British journal of cancer. 2007;97(6):785–91. Epub 2007/09/13. doi: 10.1038/sj.bjc.6603952 ; 17848913 10.1038/sj.bjc.6603952 PMC2360380

[32] Luo J, Manning BD, Cantley LC. Targeting the PI3K-Akt pathway in human cancer: rationale and promise. Cancer cell. 2003;4(4):257–62. Epub 2003/10/31. . 14585353 10.1016/s1535-6108(03)00248-4

[33] Komm BS, Kharode YP, Bodine PV, Harris HA, Miller CP, Lyttle CR. Bazedoxifene acetate: a selective estrogen receptor modulator with improved selectivity. Endocrinology. 2005;146(9):3999–4008. Epub 2005/06/18. doi: 10.1210/en.2005-0030 . 15961563 10.1210/en.2005-0030

[34] Lewiecki EM. Bazedoxifene and bazedoxifene combined with conjugated estrogens for the management of postmenopausal osteoporosis. Expert opinion on investigational drugs. 2007;16(10):1663–72. Epub 2007/10/10. doi: 10.1517/13543784.16.10.1663 . 17922629 10.1517/13543784.16.10.1663

[35] Chines AA, Komm BS. Bazedoxifene acetate: a novel selective estrogen receptor modulator for the prevention and treatment of postmenopausal osteoporosis. Drugs Today (Barc). 2009;45(7):507–20. Epub 2009/10/17 . 19834628 10.1358/dot.2009.45.7.1395293

[36] Li H, Xiao H, Lin L, Jou D, Kumari V, Lin J, et al Drug Design Targeting Protein-Protein Interactions (PPIs) Using Multiple Ligand Simultaneous Docking (MLSD) and Drug Repositioning: Discovery of Raloxifene and Bazedoxifene as Novel Inhibitors of IL-6/GP130 Interface. Journal of medicinal chemistry. 2014;57(3):632–41. Epub 2014/01/25. doi: 10.1021/jm401144z . 24456369 10.1021/jm401144z

[37] Dong S, Cheng Y, Yang J, Li J, Liu X, Wang X, et al MicroRNA expression signature and the role of microRNA-21 in the early phase of acute myocardial infarction. The Journal of biological chemistry. 2009;284(43):29514–25. Epub 2009/08/27. doi: 10.1074/jbc.M109.027896 ; 19706597 10.1074/jbc.M109.027896 PMC2785585

[38] Wei CC, Ball S, Lin L, Liu A, Fuchs JR, Li PK, et al Two small molecule compounds, LLL12 and FLLL32, exhibit potent inhibitory activity on STAT3 in human rhabdomyosarcoma cells. International journal of oncology. 2011;38(1):279–85. Epub 2010/11/27. . 21109950

[39] Liu ZL, Wang H, Liu J, Wang ZX. MicroRNA-21 (miR-21) expression promotes growth, metastasis, and chemo- or radioresistance in non-small cell lung cancer cells by targeting PTEN. Mol Cell Biochem. 2013;372(1–2):35–45. Epub 2012/09/08. doi: 10.1007/s11010-012-1443-3 . 22956424 10.1007/s11010-012-1443-3

[40] He L, Yao H, Fan LH, Liu L, Qiu S, Li X, et al MicroRNA-181b expression in prostate cancer tissues and its influence on the biological behavior of the prostate cancer cell line PC-3. Genetics and molecular research: GMR. 2013;12(2):1012–21. Epub 2013/04/25. doi: 10.4238/2013.April.2.17 . 23613247 10.4238/2013.April.2.17

[41] Iliopoulos D, Jaeger S, Hirsch H, Bulyk M, Struhl K. STAT3 activation of miR-21 and miR-181b-1 via PTEN and CYLD are part of the epigenetic switch linking inflammation to cancer. Molecular cell. 2010;39(4):493–506. doi: 10.1016/j.molcel.2010.07.023 20797623 10.1016/j.molcel.2010.07.023 PMC2929389

[42] Niu G, Wright KL, Huang M, Song L, Haura E, Turkson J, et al Constitutive Stat3 activity up-regulates VEGF expression and tumor angiogenesis. Oncogene. 2002;21(13):2000–8. doi: 10.1038/sj.onc.1205260 . 11960372 10.1038/sj.onc.1205260

[43] Wei D, Le X, Zheng L, Wang L, Frey JA, Gao AC, et al Stat3 activation regulates the expression of vascular endothelial growth factor and human pancreatic cancer angiogenesis and metastasis. Oncogene. 2003;22(3):319–29. Epub 2003/01/25. doi: 10.1038/sj.onc.1206122 . 12545153 10.1038/sj.onc.1206122

[44] Xie T, Wei D, Liu M, Gao A, Ali-Osman F, Sawaya R, et al Stat3 activation regulates the expression of matrix metalloproteinase-2 and tumor invasion and metastasis. Oncogene. 2004;23(20):3550–60. doi: 10.1038/sj.onc.1207383 15116091 10.1038/sj.onc.1207383

[45] Kitamura Y, Morita I, Nihei Z, Mishima Y, Murota S. Effect of IL-6 on tumor cell invasion of vascular endothelial monolayers. Surgery today. 1997;27(6):534–41. Epub 1997/01/01. . 9306547 10.1007/BF02385807

[46] Song L, Rawal B, Nemeth JA, Haura EB. JAK1 activates STAT3 activity in non-small-cell lung cancer cells and IL-6 neutralizing antibodies can suppress JAK1-STAT3 signaling. Molecular cancer therapeutics. 2011;10(3):481–94. Epub 2011/01/11. doi: 10.1158/1535-7163.MCT-10-0502 . 21216930 10.1158/1535-7163.MCT-10-0502 PMC4084653

[47] Pu YS, Hour TC, Chuang SE, Cheng AL, Lai MK, Kuo ML. Interleukin-6 is responsible for drug resistance and anti-apoptotic effects in prostatic cancer cells. The Prostate. 2004;60(2):120–9. Epub 2004/05/27. doi: 10.1002/pros.20057 . 15162378 10.1002/pros.20057

[48] Trikha M, Corringham R, Klein B, Rossi JF. Targeted anti-interleukin-6 monoclonal antibody therapy for cancer: a review of the rationale and clinical evidence. Clinical cancer research: an official journal of the American Association for Cancer Research. 2003;9(13):4653–65. Epub 2003/10/29. ; 14581334 PMC2929399

[49] Fizazi K, De Bono JS, Flechon A, Heidenreich A, Voog E, Davis NB, et al Randomised phase II study of siltuximab (CNTO 328), an anti-IL-6 monoclonal antibody, in combination with mitoxantrone/prednisone versus mitoxantrone/prednisone alone in metastatic castration-resistant prostate cancer. Eur J Cancer. 2012;48(1):85–93. Epub 2011/12/02. doi: 10.1016/j.ejca.2011.10.014 . 22129890 10.1016/j.ejca.2011.10.014

[50] Voorhees PM, Manges RF, Sonneveld P, Jagannath S, Somlo G, Krishnan A, et al A phase 2 multicentre study of siltuximab, an anti-interleukin-6 monoclonal antibody, in patients with relapsed or refractory multiple myeloma. British journal of haematology. 2013;161(3):357–66. Epub 2013/02/26. doi: 10.1111/bjh.12266 . 23432640 10.1111/bjh.12266 PMC5837861

[51] Rossi JF, Negrier S, James ND, Kocak I, Hawkins R, Davis H, et al A phase I/II study of siltuximab (CNTO 328), an anti-interleukin-6 monoclonal antibody, in metastatic renal cell cancer. British journal of cancer. 2010;103(8):1154–62. Epub 2010/09/03. doi: 10.1038/sj.bjc.6605872 ; 20808314 10.1038/sj.bjc.6605872 PMC2967052

[52] Saleh AZ, Greenman KL, Billings S, Van Vranken DL, Krolewski JJ. Binding of madindoline A to the extracellular domain of gp130. Biochemistry. 2005;44(32):10822–7. Epub 2005/08/10. doi: 10.1021/bi050439+ . 16086584 10.1021/bi050439+

[53] Kharode Y, Bodine PV, Miller CP, Lyttle CR, Komm BS. The pairing of a selective estrogen receptor modulator, bazedoxifene, with conjugated estrogens as a new paradigm for the treatment of menopausal symptoms and osteoporosis prevention. Endocrinology. 2008;149(12):6084–91. Epub 2008/08/16. doi: 10.1210/en.2008-0817 . 18703623 10.1210/en.2008-0817

[54] Li RF, Gupta M, McCluggage WG, Ronnett BM. Embryonal rhabdomyosarcoma (botryoid type) of the uterine corpus and cervix in adult women: report of a case series and review of the literature. The American journal of surgical pathology. 2013;37(3):344–55. Epub 2013/01/26. doi: 10.1097/PAS.0b013e31826e0271 . 23348207 10.1097/PAS.0b013e31826e0271

[55] Greenberg JA, Somme S, Russnes HE, Durbin AD, Malkin D. The estrogen receptor pathway in rhabdomyosarcoma: a role for estrogen receptor-beta in proliferation and response to the antiestrogen 4'OH-tamoxifen. Cancer research. 2008;68(9):3476–85. Epub 2008/05/03. doi: 10.1158/0008-5472.CAN-07-3046 . 18451176 10.1158/0008-5472.CAN-07-3046

[56] Lavie O, Barnett-Griness O, Narod SA, Rennert G. The risk of developing uterine sarcoma after tamoxifen use. International journal of gynecological cancer: official journal of the International Gynecological Cancer Society. 2008;18(2):352–6. Epub 2008/03/13. doi: 10.1111/j.1525-1438.2007.01025.x . 18334013 10.1111/j.1525-1438.2007.01025.x

[57] Rieck GC, Freites ON, Williams S. Is tamoxifen associated with high-risk endometrial carcinomas? A retrospective case series of 196 women with endometrial cancer. Journal of obstetrics and gynaecology: the journal of the Institute of Obstetrics and Gynaecology. 2005;25(1):39–41. Epub 2005/09/09. doi: 10.1080/01443610400024740 . 16147692 10.1080/01443610400024740

[58] Archer DF, Pinkerton JV, Utian WH, Menegoci JC, de Villiers TJ, Yuen CK, et al Bazedoxifene, a selective estrogen receptor modulator: effects on the endometrium, ovaries, and breast from a randomized controlled trial in osteoporotic postmenopausal women. Menopause. 2009;16(6):1109–15. Epub 2009/06/23. doi: 10.1097/gme.0b013e3181a818db . 19543129 10.1097/gme.0b013e3181a818db

[59] Palacios S, Silverman SL, de Villiers TJ, Levine AB, Goemaere S, Brown JP, et al A 7-year randomized, placebo-controlled trial assessing the long-term efficacy and safety of bazedoxifene in postmenopausal women with osteoporosis: effects on bone density and fracture. Menopause. 2015 Epub 2015/02/11. doi: 10.1097/GME.0000000000000419 . 25668306 10.1097/GME.0000000000000419

[60] Lewis-Wambi JS, Kim H, Curpan R, Grigg R, Sarker MA, Jordan VC. The selective estrogen receptor modulator bazedoxifene inhibits hormone-independent breast cancer cell growth and down-regulates estrogen receptor alpha and cyclin D1. Molecular pharmacology. 2011;80(4):610–20. Epub 2011/07/09. doi: 10.1124/mol.111.072249 ; 21737572 10.1124/mol.111.072249 PMC3187528

[61] Song Y, Santen RJ, Wang JP, Yue W. Inhibitory effects of a bazedoxifene/conjugated equine estrogen combination on human breast cancer cells in vitro. Endocrinology. 2013;154(2):656–65. Epub 2012/12/21. doi: 10.1210/en.2012-2038 . 23254198 10.1210/en.2012-2038

