# Supplementary material for: Repositioning Bazedoxifene as a novel IL-6/GP130 signaling antagonist for human rhabdomyosarcoma therapy
Source: PLoS One. 2017 Jul 3;12(7):e0180297. doi: 10.1371/journal.pone.0180297 (PMC5495564; doi:10.1371/journal.pone.0180297)
Supplement: S1 Fig — RH5 rhabdomyosarcoma cells with lower P-STAT3 expression was cultured in FBS free media for 24 hours. Cells were pretreated with Bazedoxifene at the indicated concentration for 8 hours, and then IL-6 (50ng/ml) was added for 30 minutes. The expression level of P-STAT3 (Y705) was assessed by Western blot analysis with GAPDH as loading control. (PPTX) [file pone.0180297.s001.pptx]

## Slide 1
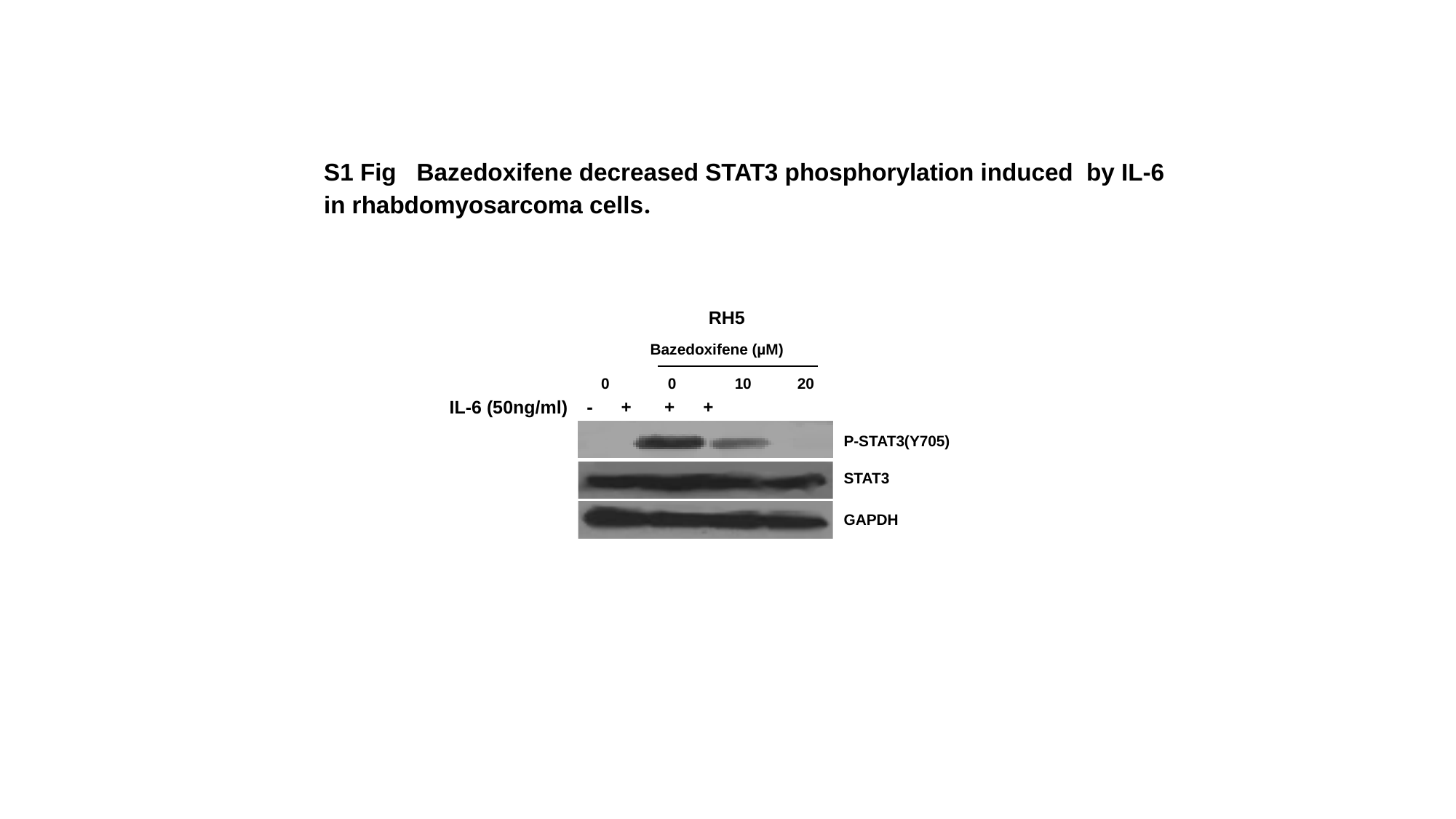

S1 Fig Bazedoxifene decreased STAT3 phosphorylation induced by IL-6 in rhabdomyosarcoma cells.
RH5
Bazedoxifene (µM)
 0 0 10 20
IL-6 (50ng/ml) - + + +
P-STAT3(Y705)
STAT3
GAPDH
