# Supplementary material for: Repositioning Bazedoxifene as a novel IL-6/GP130 signaling antagonist for human rhabdomyosarcoma therapy
Source: PLoS One. 2017 Jul 3;12(7):e0180297. doi: 10.1371/journal.pone.0180297 (PMC5495564; doi:10.1371/journal.pone.0180297)
Supplement: S2 Fig — RD rhabdomyosarcoma cells were treated with Bazedoxifene 15μM for 16 hours. Then cell lysates were subjected to immunoprecipitation and the expression level of GP130, IL-6R, JAK1, and STAT3 was evaluated using Western blot analysis with GAPDH as loading control. (PPTX) [file pone.0180297.s002.pptx]

## Slide 1
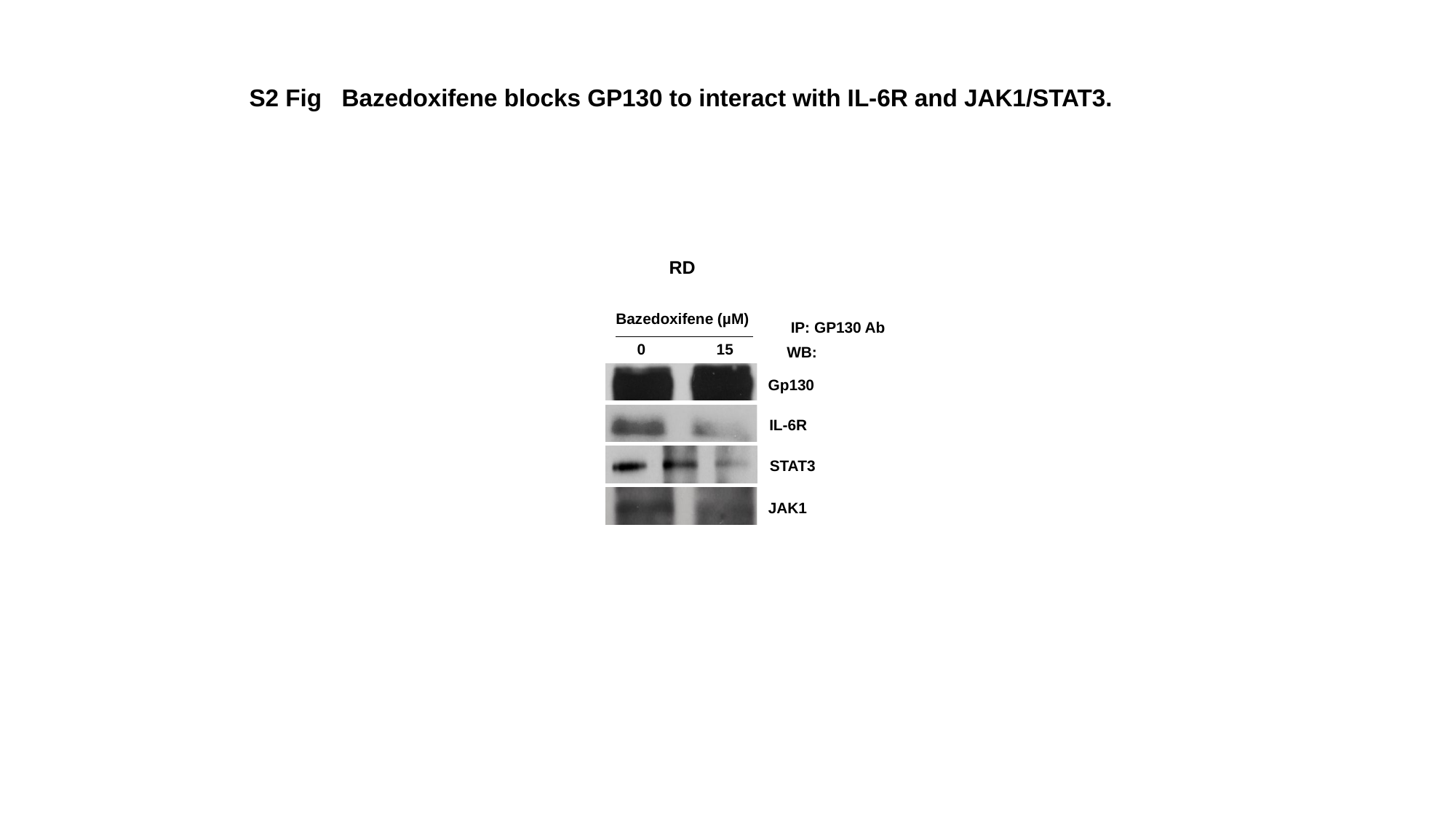

S2 Fig Bazedoxifene blocks GP130 to interact with IL-6R and JAK1/STAT3.
RD
Bazedoxifene (µM)
IP: GP130 Ab
 0 15
WB:
Gp130
IL-6R
STAT3
JAK1
