# Supplementary material for: Repositioning Bazedoxifene as a novel IL-6/GP130 signaling antagonist for human rhabdomyosarcoma therapy
Source: PLoS One. 2017 Jul 3;12(7):e0180297. doi: 10.1371/journal.pone.0180297 (PMC5495564; doi:10.1371/journal.pone.0180297)
Supplement: S3 Fig — The human ER-β siRNA or negative control siRNA was transfected into RD (50nM), RH28 (100nM) and RH30 (50nM) cells using Lipofectamine2000. A, Western blot assay was used to detect the expression of ER-β in the transfected cells to confirm the transfection efficacy. B, MTT assay was conducted to detect cell viability of the transfected rhabdomyosarcoma cells. (PPTX) [file pone.0180297.s003.pptx]

## Slide 1
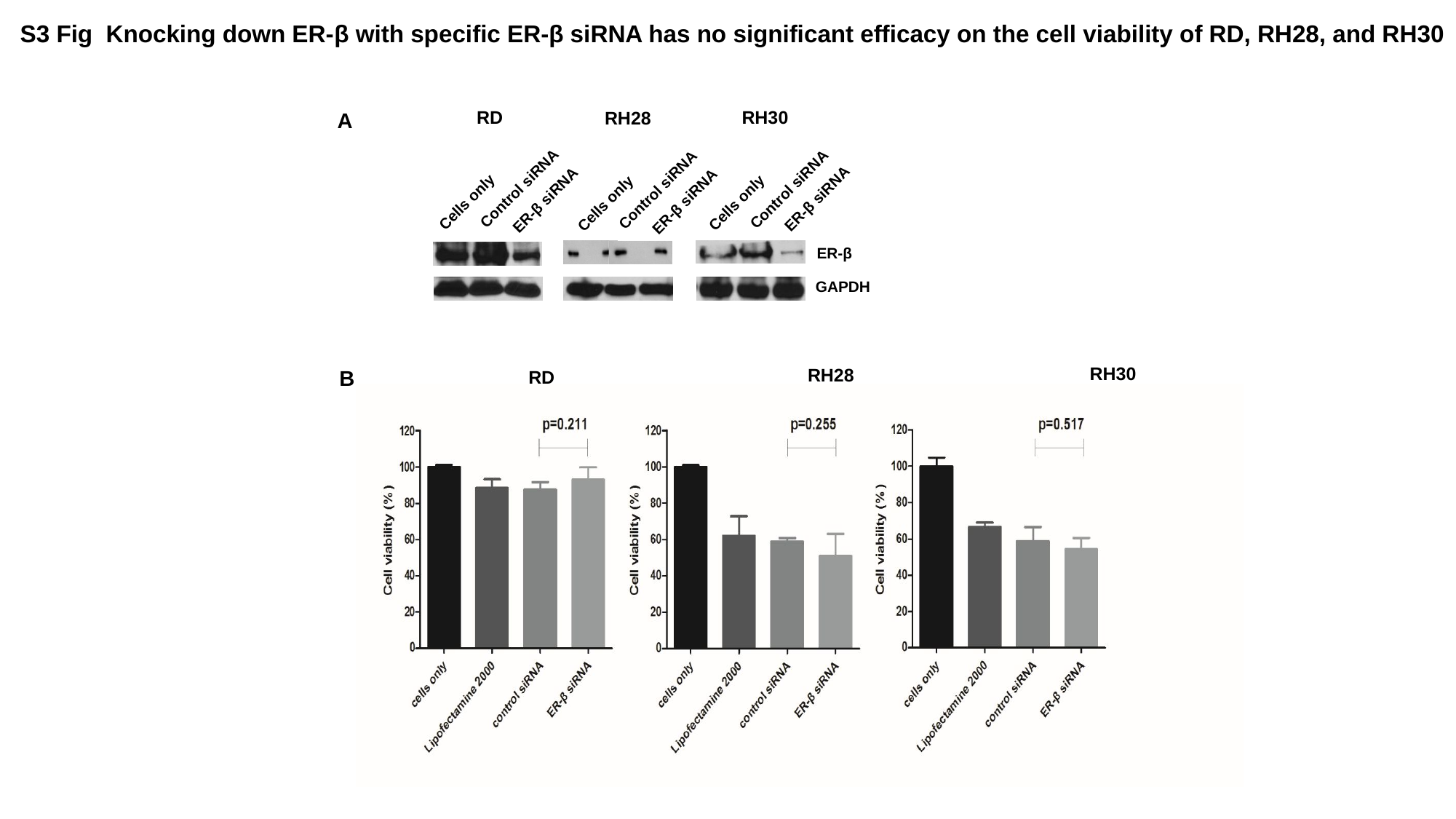

S3 Fig Knocking down ER-β with specific ER-β siRNA has no significant efficacy on the cell viability of RD, RH28, and RH30
RD
RH30
RH28
A
Control siRNA
ER-β siRNA
Cells only
Control siRNA
ER-β siRNA
Cells only
Control siRNA
ER-β siRNA
Cells only
ER-β
GAPDH
RH30
RH28
B
RD
