# Supplementary material for: Repositioning Bazedoxifene as a novel IL-6/GP130 signaling antagonist for human rhabdomyosarcoma therapy
Source: PLoS One. 2017 Jul 3;12(7):e0180297. doi: 10.1371/journal.pone.0180297 (PMC5495564; doi:10.1371/journal.pone.0180297)
Supplement: S1 Table — (PPTX) [file pone.0180297.s004.pptx]

## Slide 1
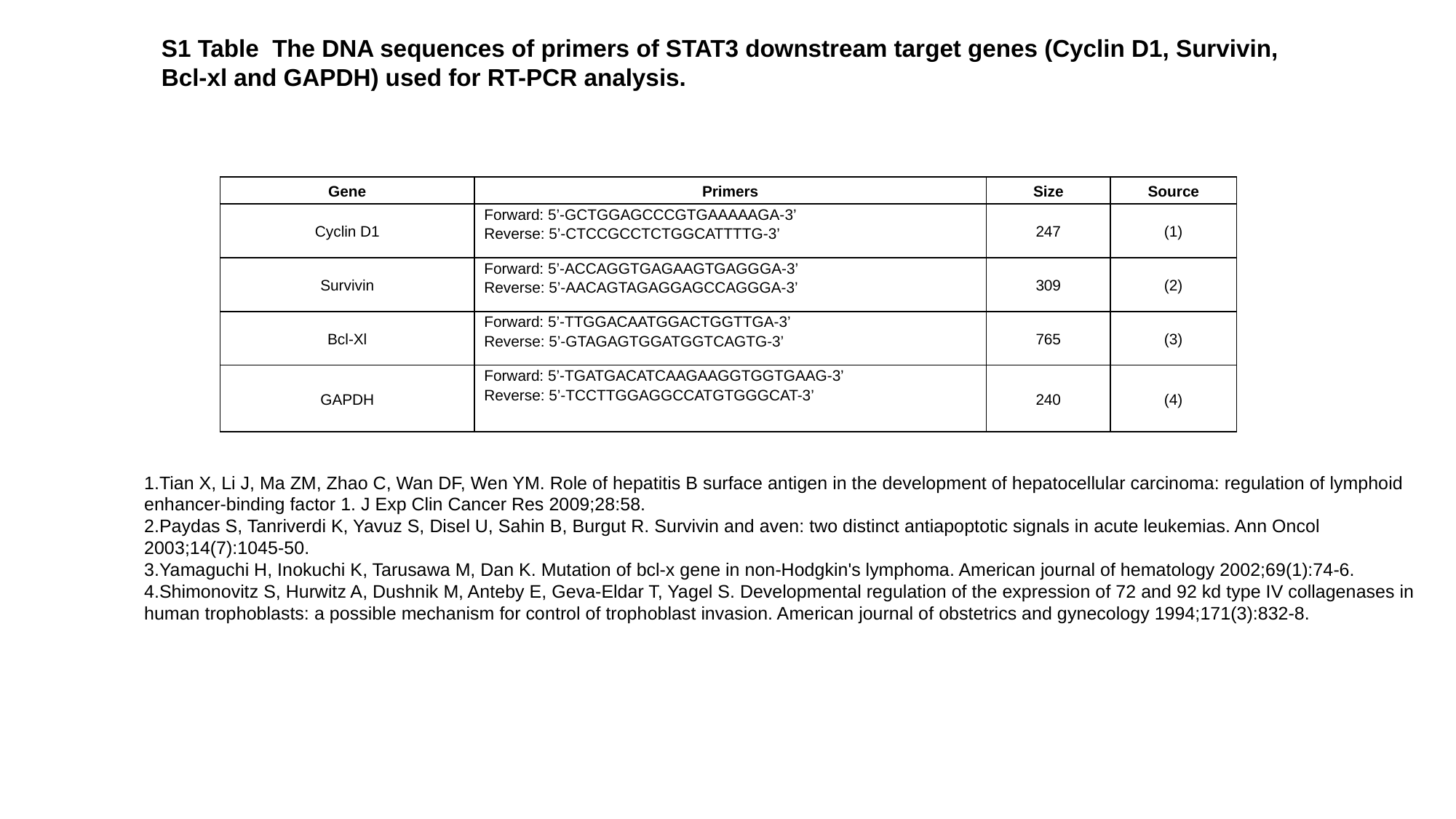

S1 Table The DNA sequences of primers of STAT3 downstream target genes (Cyclin D1, Survivin, Bcl-xl and GAPDH) used for RT-PCR analysis.
| Gene | Primers | Size | Source |
| --- | --- | --- | --- |
| Cyclin D1 | Forward: 5’-GCTGGAGCCCGTGAAAAAGA-3’ Reverse: 5’-CTCCGCCTCTGGCATTTTG-3’ | 247 | (1) |
| Survivin | Forward: 5’-ACCAGGTGAGAAGTGAGGGA-3’ Reverse: 5’-AACAGTAGAGGAGCCAGGGA-3’ | 309 | (2) |
| Bcl-Xl | Forward: 5’-TTGGACAATGGACTGGTTGA-3’ Reverse: 5’-GTAGAGTGGATGGTCAGTG-3’ | 765 | (3) |
| GAPDH | Forward: 5’-TGATGACATCAAGAAGGTGGTGAAG-3’ Reverse: 5’-TCCTTGGAGGCCATGTGGGCAT-3’ | 240 | (4) |
1.Tian X, Li J, Ma ZM, Zhao C, Wan DF, Wen YM. Role of hepatitis B surface antigen in the development of hepatocellular carcinoma: regulation of lymphoid enhancer-binding factor 1. J Exp Clin Cancer Res 2009;28:58.
2.Paydas S, Tanriverdi K, Yavuz S, Disel U, Sahin B, Burgut R. Survivin and aven: two distinct antiapoptotic signals in acute leukemias. Ann Oncol 2003;14(7):1045-50.
3.Yamaguchi H, Inokuchi K, Tarusawa M, Dan K. Mutation of bcl-x gene in non-Hodgkin's lymphoma. American journal of hematology 2002;69(1):74-6.
4.Shimonovitz S, Hurwitz A, Dushnik M, Anteby E, Geva-Eldar T, Yagel S. Developmental regulation of the expression of 72 and 92 kd type IV collagenases in human trophoblasts: a possible mechanism for control of trophoblast invasion. American journal of obstetrics and gynecology 1994;171(3):832-8.
